# Prevalence and risk of mental disorders in the perinatal period among migrant women: a systematic review and meta-analysis

**DOI:** 10.1007/s00737-017-0723-z

**Published:** 2017-04-08

**Authors:** Fraser M Anderson, Stephani L Hatch, Carla Comacchio, Louise M Howard

**Affiliations:** 1 Section of Women’s Mental Health, IOPPN, King’s College London, Box P031, David Goldberg Centre, 16 De Crespigny Park, London, SE5 8AF UK; 2 Department of Psychological Medicine, IOPPN, King’s College London, London, UK; 3 Department of Neurosciences, Biomedicine and Movement Sciences, University of Verona, Verona, Italy

**Keywords:** Perinatal mental health, Migrant women, Refugees, Asylum-seekers, Postpartum depression, Antenatal depression

## Abstract

This study was conducted in order to evaluate the prevalence and risk of mental disorders in the perinatal period among migrant women. Six databases (including MEDLINE) were searched from inception to October 19th, 2015, in addition to citation tracking. Studies were eligible if mental disorders were assessed with validated tools during pregnancy and up to 1 year postpartum among women born outside of the study country. Of 3241 abstracts screened, 53 met the inclusion criteria for the review. Only three studies investigated a mental disorder other than depression. Unadjusted odds ratios were pooled using random effects meta-analysis for elevated depression symptoms during pregnancy (*n* = 12) and the postpartum (*n* = 24), stratified by study country due to heterogeneity. Studies from Canada found an increased risk for antenatal (OR = 1.86, 95% CIs 1.32–2.62) and postnatal elevated depression symptoms (OR = 1.98, 95% CIs 1.57–2.49) associated with migrant status. Studies from the USA found a decreased risk of antenatal elevated depression symptoms (OR = 0.71, 95% CIs 0.51–0.99), and studies from the USA and Australia found no association between migrant status and postnatal elevated depression symptoms. Low social support, minority ethnicity, low socioeconomic status, lack of proficiency in host country language and refugee or asylum-seeking status all put migrant populations at increased risk of perinatal mental disorders.

**Electronic supplementary material:**

The online version of this article (doi:10.1007/s00737-017-0723-z) contains supplementary material, which is available to authorized users.

## Introduction

In the general population, mental health disorders during the perinatal period (pregnancy and up to 1 year postpartum) appear to be common with a study in the USA finding a 12-month prevalence of 25.3% for pregnant women and 27.5% for postpartum women (Vesga-Lopez et al. 2008). Mental disorders during pregnancy and the postpartum period are associated with adverse outcomes for both the mother and the child (Bauer et al. 2014). There is some evidence for moderation of this association, whereby it is stronger in low- and middle-income countries (LMICs) and generally among low socioeconomic groups (Stein et al. 2014). As migrant women are more likely to be in poverty, and most global migration is from LMICs (United Nations 2013), we may expect worse adverse child outcomes associated with perinatal mental disorders among migrant women.

As the proportion of people living outside of their country of birth increases globally, recognising the health needs of migrants is increasingly important. We use the term ‘migrant’ to refer to all people living outside of their country of birth, including refugees, asylum-seekers, economic migrants and all other reasons for migration and legal statuses. Migrant populations are increasingly composed of women, often of childbearing age, and so understanding the health of migrant women in the perinatal period is particularly urgent (United Nations 2013). There are numerous contradictory findings in the field of migrant health, with a ‘healthy migrant effect’ observed for particular outcomes and yet increased vulnerability of migrants to other outcomes, in both mental and physical health (Rechel et al. 2013). There is evidence in general population studies of an initial healthy migrant effect for mental and physical health that then deteriorates over time, to show same or worse health than non-migrants, despite improvements in socioeconomic status (De Maio and Kemp 2010). Contradictory evidence exists for maternal and child health, with a systematic review finding that although perinatal health outcomes were not consistently poorer among migrant women in general, geographical origin was important, with Asian and African migrants at increased risk of foetus-infant mortality and preterm birth than the majority receiving populations of western industrialized countries (Gagnon et al. 2009).

While there is a substantial amount of research on maternal and child physical outcomes among migrants, less is known about the association between migrant status and maternal mental health during the perinatal period. Despite the evidence for risk of mental health problems among migrants in general being very mixed, it appears that mental disorders during the perinatal period may be higher among migrant than non-migrant women. A review found that postnatal depression may affect up to 42% of migrant women compared to 10–15% of native-born women, and antenatal depression appears to be higher as well (Collins et al. 2011b; Miszkurka et al. 2012b). A recent systematic review investigated the prevalence of perinatal mental disorders in migrant women from LMICs (Fellmeth et al. 2016); however, there has been no systematic review examining the prevalence and risk of perinatal mental disorders in migrant women from all settings. Furthering the evidence for prevalence and risk of perinatal mental disorders among migrant women may help to inform clinical practice and develop interventions that meet the specific needs of migrant women.

The objectives of this systematic review were as follows:

1. To describe the state of the literature related to perinatal mental disorders among migrant women
2. To determine the prevalence of perinatal mental disorders among migrant women
3. To determine the risk of perinatal mental disorders among migrant women compared to non-migrant women
4. To identify risk factors for perinatal mental disorders among migrant women

## Methods

The review followed the PRISMA guidelines and the protocol was registered with the prospective register of systematic reviews (PROSPERO; registration number CRD42015016262).

### Sources

EMBASE, MEDLINE, PsycINFO, CINAHL, Maternal and Infant Care and Cochrane Register of Controlled Trials (CENTRAL) were searched from inception for published peer-reviewed literature. The search was run on 19th October 2015. Forward and backward citation tracking of included papers was conducted to identify any relevant papers missed in the search. The search strategy included terms related to the perinatal period (pregnancy and postpartum), migration and mental disorders and all relevant synonyms. A combination of MeSH terms and free-text searches was used (see Supplementary material for a full list of search terms).

### Study selection

Studies were eligible for inclusion if they assessed antenatal (any point in pregnancy) or postpartum (up to a year after delivery, which is the typical timeframe for both studies and clinical practice addressing the postpartum period) mental disorders in migrant women (born outside of the study country). Studies that included migrant women only were included in prevalence estimates but not in the estimates of risk as this required non-migrant (born in study country) controls. Diagnostic and validated screening measures for mental disorders were accepted. Studies were not eligible if they used a proxy measure for migrant status, e.g. ethnicity. Cohort, case-control, cross-sectional and intervention studies (with appropriate baseline data) were included in the review. Only published, peer-reviewed English language papers were eligible.

All of the citations from the searches were downloaded into Endnote™, and duplicate citations were removed. Two reviewers (FA and CC) independently screened the titles and abstracts of all the citations in the database for relevance to the review. Full text articles for all relevant citations identified by the reviewers were obtained. All full text articles were assessed for eligibility using a checklist of the inclusion and exclusion criteria for the review, independently by two reviewers (FA and CC). All excluded articles were retained with their reason for exclusion noted. Any disagreements between reviewers were discussed, and agreement was sought. Where there was insufficient information reported in the article to assess eligibility, the authors were contacted to provide the missing information. Where it appeared that the relevant data had been collected, e.g. country of birth and mental disorder during pregnancy, but the disaggregated results were not reported, authors were contacted to provide the raw data for analysis. One reviewer extracted the relevant information from the articles using a piloted data extraction form developed for this review. The risk of bias in the included studies was assessed using a piloted risk of bias tool adapted from previous quality assessment tools (Downs and Black 1998; MacLehose et al. 2000; Trevillion et al. 2012; Wells et al. 2009) and the Critical Appraisal Skills Programme (CASP) checklist (Critical Appraisal Skills Programme (CASP) 2014). The tool assessed the domains of measurement bias and selection bias. Two reviewers (FA and CC) independently rated the studies for risk of bias, with any disagreements discussed.

### Analysis

Studies were grouped by mental disorder and time period (antenatal or postpartum). Heterogeneity between studies was explored using the *I* ^2^ statistic. Pooled prevalence estimates with 95% confidence intervals were calculated using random effects meta-analysis to allow for expected heterogeneity between different study populations. Pooled estimates of odds ratios with 95% confidence intervals were calculated to look at risk of disorder among migrant women compared to non-migrant women using random effects meta-analysis. All of the odds ratios presented are unadjusted (calculated from raw data extracted from papers or from author contact) in order for comparison between studies, as different studies adjusted for different variables.

Sensitivity analyses (excluding studies with high risk of bias, stratifying by measure of disorder and stratifying by study country) were performed to explore heterogeneity of the studies. Despite planning sensitivity analyses to disaggregate the migrant group by country of birth, legal immigration status, reason for migration and other relevant factors (e.g. age and ethnicity), these analyses were not possible due to the data from most studies not including an appropriate level of detail. All statistical analyses were conducted using Stata 13 (2013).

## Results

**Fig. 1:**
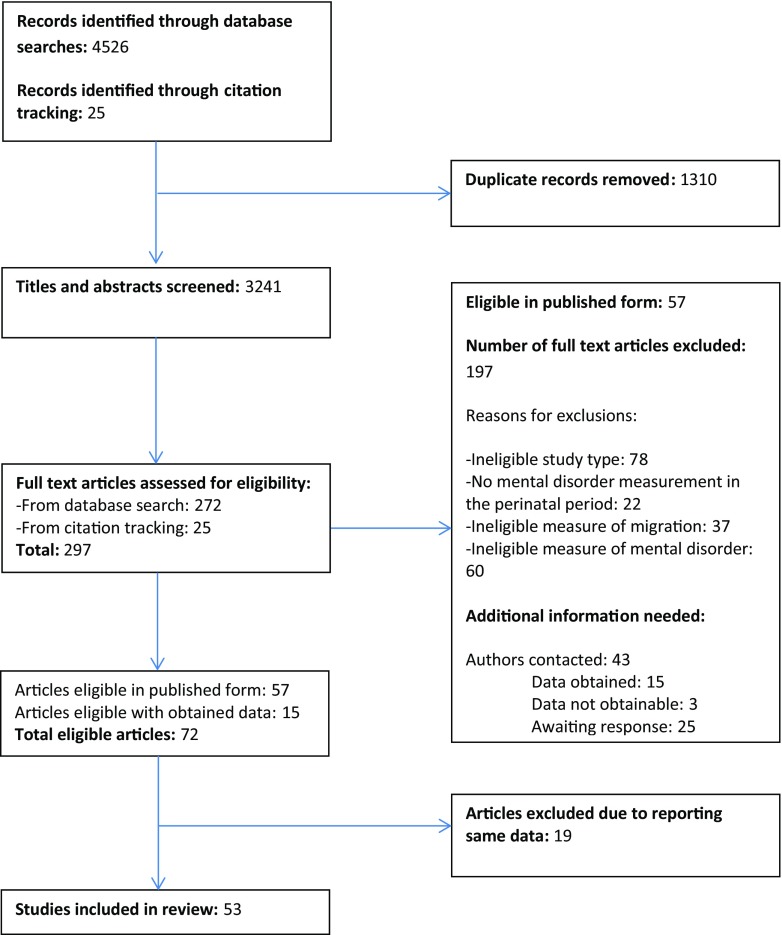
Flow diagram of the study selection process

A flow diagram of the study selection process is shown in Fig. 1. A total of 3241 unique references were screened, with a total of 297 full text articles assessed for eligibility. A total of 53 original studies were included in the review; reasons for exclusion are given in Fig. 1. The studies included in the review are detailed in the table of studies (see Supplementary material).

All of the studies were conducted in high-income countries. The countries with the highest numbers of studies were Canada, 15 studies (Ballantyne et al. 2013; Dennis et al. 2004; Gagnon et al. 2013; Ganann et al. 2012; Lanes et al. 2011; McDonald et al. 2013; Mechakra-Tahiri et al. 2007; Minde et al. 2001; Miszkurka et al. 2010; Peer et al. 2013; Stewart et al. 2008, 2012; Sword et al. 2006; Van Lieshout et al. 2011; Zelkowitz et al. 2008); the USA, 14 studies (Balbierz et al. 2014; Connelly et al. 2013; Davila et al. 2009; Diaz et al. 2007; Elo and Culhane 2010; Fleuriet and Sunil 2014; Fortner et al. 2011; Goyal et al. 2006; Harrison and Sidebottom 2009; Heilemann et al. 2004; Huang et al. 2007; Luecken et al. 2013; Valentine et al. 2011; Yeung and Schwartz 1986); Australia, 7 studies (Bandyopadhyay et al. 2010; Eastwood et al. 2011; Milgrom et al. 2008; Shafiei et al. 2015; Small et al. 2003; Tran et al. 2002; Yelland et al. 2010); and Taiwan, China, 6 studies (Chen et al. 2013; Chen et al. 2012; Chien et al. 2012; Huang and Mathers 2008; Hung et al. 2012; Tsao et al. 2014).

Thirty-one studies were cross-sectional surveys (Abbott and Williams 2006; Balestrieri et al. 2012; Ballantyne et al. 2013; Chen et al. 2012; Chien et al. 2012; Choi et al. 2012; Davila et al. 2009; Dennis et al. 2004; Eastwood et al. 2011; Elo and Culhane 2010; Fisch et al. 1997; Fleuriet and Sunil 2014; Ganann et al. 2012; Glasser et al. 2000; Goyal et al. 2006; Harrison and Sidebottom 2009; Heilemann et al. 2004; Huang and Mathers 2008; Hung et al. 2012; Lanes et al. 2011; Mechakra-Tahiri et al. 2007; Minde et al. 2001; Miszkurka et al. 2010; Peer et al. 2013; Ratcliff et al. 2015; Shafiei et al. 2015; Small et al. 2003; Stewart et al. 2008; Sword et al. 2006; Yelland et al. 2010; Yeung and Schwartz 1986), 20 were prospective cohorts (Bandyopadhyay et al. 2010; Bjerke et al. 2008; Chen et al. 2013; Diaz et al. 2007; Escribà-Agüir et al. 2013; Fortner et al. 2011; Gagnon et al. 2013; Huang et al. 2007; Jayaweera and Quigley 2010; Luecken et al. 2013; McDonald et al. 2013; Milgrom et al. 2008; Rudman et al. 2008; Stewart et al. 2012; Tran et al. 2002; Tsao et al. 2014; Valentine et al. 2011; Van Lieshout et al. 2011; Yoshida et al. 1997; Zelkowitz et al. 2008) and two were randomized controlled trials (Balbierz et al. 2014; Connelly et al. 2013).

Fifty studies looked at depression alone (Abbott and Williams 2006; Balbierz et al. 2014; Balestrieri et al. 2012; Ballantyne et al. 2013; Bandyopadhyay et al. 2010; Bjerke et al. 2008; Chen et al. 2012, 2013; Chien et al. 2012; Choi et al. 2012; Connelly et al. 2013; Davila et al. 2009; Dennis et al. 2004; Diaz et al. 2007; Eastwood et al. 2011; Elo and Culhane 2010; Escribà-Agüir et al. 2013; Fisch et al. 1997; Fleuriet and Sunil 2014; Fortner et al. 2011; Ganann et al. 2012; Glasser et al. 2000; Goyal et al. 2006; Harrison and Sidebottom 2009; Heilemann et al. 2004; Huang and Mathers 2008; Huang et al. 2007; Hung et al. 2012; Jayaweera and Quigley 2010; Lanes et al. 2011; Luecken et al. 2013; McDonald et al. 2013; Mechakra-Tahiri et al. 2007; Milgrom et al. 2008; Minde et al. 2001; Miszkurka et al. 2010; Peer et al. 2013; Ratcliff et al. 2015; Rudman et al. 2008; Shafiei et al. 2015; Small et al. 2003; Stewart et al. 2008, 2012; Sword et al. 2006; Tran et al. 2002; Tsao et al. 2014; Valentine et al. 2011; Van Lieshout et al. 2011; Yoshida et al. 1997; Zelkowitz et al. 2008), one study depression and anxiety (Yelland et al. 2010), one study depression and posttraumatic stress disorder (PTSD) (Gagnon et al. 2013) and one study looked at any DSM-III diagnosis (Yeung and Schwartz 1986).

Six studies measured mental disorder symptoms in both pregnancy and the postpartum period with data presented separately, at least two time-points (Diaz et al. 2007; Heilemann et al. 2004; McDonald et al. 2013; Rudman et al. 2008; Tsao et al. 2014; Zelkowitz et al. 2008). Three studies measured mental disorder symptoms combining women who were pregnant and in the postpartum period (Connelly et al. 2013; Davila et al. 2009; Tran et al. 2002). Thirty-three studies investigated the postpartum period only (Abbott and Williams 2006; Balbierz et al. 2014; Ballantyne et al. 2013; Bandyopadhyay et al. 2010; Bjerke et al. 2008; Chen et al. 2012, 2013; Chien et al. 2012; Choi et al. 2012; Dennis et al. 2004; Eastwood et al. 2011; Fisch et al. 1997; Gagnon et al. 2013; Ganann et al. 2012; Glasser et al. 2000; Goyal et al. 2006; Huang and Mathers 2008; Huang et al. 2007; Hung et al. 2012; Jayaweera and Quigley 2010; Lanes et al. 2011; Mechakra-Tahiri et al. 2007; Milgrom et al. 2008; Minde et al. 2001; Shafiei et al. 2015; Small et al. 2003; Stewart et al. 2008; Stewart et al. 2012; Sword et al. 2006; Valentine et al. 2011; Van Lieshout et al. 2011; Yelland et al. 2010; Yoshida et al. 1997) and 11 studies looked only at pregnancy (Balestrieri et al. 2012; Elo and Culhane 2010; Escribà-Agüir et al. 2013; Fleuriet and Sunil 2014; Fortner et al. 2011; Harrison and Sidebottom 2009; Luecken et al. 2013; Miszkurka et al. 2010; Peer et al. 2013; Ratcliff et al. 2015; Yeung and Schwartz 1986). Studies with women in the postpartum period ranged from 1 week to 1 year postdelivery.

### Risk of bias

Studies were given a rating of high, medium or low risk of measurement and selection bias, using the risk of bias tool developed for the review. Only two studies (Yeung and Schwartz 1986; Yoshida et al. 1997) scored low on risk of measurement bias, as they used a diagnostic instrument to measure mental disorder, whereas all the other studies used validated screening measures and scored medium. Six studies scored low on risk of selection bias (Connelly et al. 2013; Ganann et al. 2012; Harrison and Sidebottom 2009; Huang et al. 2007; Jayaweera and Quigley 2010; Lanes et al. 2011), 23 medium (Abbott and Williams 2006; Balbierz et al. 2014; Balestrieri et al. 2012; Ballantyne et al. 2013; Bandyopadhyay et al. 2010; Dennis et al. 2004; Eastwood et al. 2011; Elo and Culhane 2010; Escribà-Agüir et al. 2013; Gagnon et al. 2013; Glasser et al. 2000; McDonald et al. 2013; Mechakra-Tahiri et al. 2007; Milgrom et al. 2008; Miszkurka et al. 2010; Rudman et al. 2008; Small et al. 2003; Stewart et al. 2008, 2012; Sword et al. 2006; Tran et al. 2002; Van Lieshout et al. 2011; Zelkowitz et al. 2008) and 24 high (Bjerke et al. 2008; Chen et al. 2012, 2013; Chien et al. 2012; Choi et al. 2012; Davila et al. 2009; Diaz et al. 2007; Fisch et al. 1997; Fleuriet and Sunil 2014; Fortner et al. 2011; Goyal et al. 2006; Heilemann et al. 2004; Huang and Mathers 2008; Hung et al. 2012; Luecken et al. 2013; Minde et al. 2001; Peer et al. 2013; Ratcliff et al. 2015; Shafiei et al. 2015; Tsao et al. 2014; Valentine et al. 2011; Yelland et al. 2010; Yeung and Schwartz 1986; Yoshida et al. 1997). The main reasons for studies scoring high on selection bias were biased sampling techniques such as volunteer or convenience samples, only including one ethnic group, and exclusion of participants due to language spoken. Inclusion and exclusion criteria of studies are reported in the table of studies (see Supplementary material).

### Prevalence of depression among migrant women

The measures used for depression are shown in Table 1. As almost all of the studies used screening tools with cut-off scores rather than diagnostic measures, prevalence and risk estimates are reported for elevated depression symptoms (indicating probable depression) rather than depression diagnosis.

**Table 1:** Measures of depression used by included studies

Measure of depression	Studies
Edinburgh Postnatal Depression Scale (EPDS) (Cox et al. 1987)	35 (Abbott and Williams 2006; Balbierz et al. 2014; Balestrieri et al. 2012; Bandyopadhyay et al. 2010; Bjerke et al. 2008; Chen et al. 2012, 2013; Chien et al. 2012; Choi et al. 2012; Connelly et al. 2013; Dennis et al. 2004; Eastwood et al. 2011; Escribà-Agüir et al. 2013; Fisch et al. 1997; Fortner et al. 2011; Gagnon et al. 2013; Ganann et al. 2012; Glasser et al. 2000; Huang and Mathers 2008; Lanes et al. 2011; Luecken et al. 2013; McDonald et al. 2013; Milgrom et al. 2008; Minde et al. 2001; Peer et al. 2013; Ratcliff et al. 2015; Rudman et al. 2008; Shafiei et al. 2015; Small et al. 2003; Stewart et al. 2008; Stewart et al. 2012; Sword et al. 2006; Tran et al. 2002; Tsao et al. 2014; Zelkowitz et al. 2008)
Center for Epidemiologic Studies Depression Scale (CES-D) (Radloff 1977)	9 (Ballantyne et al. 2013; Davila et al. 2009; Diaz et al. 2007; Elo and Culhane 2010; Heilemann et al. 2004; Huang et al. 2007; Mechakra-Tahiri et al. 2007; Miszkurka et al. 2010; Van Lieshout et al. 2011)
Patient Health Questionnaire (PHQ-9)	2 (Fleuriet and Sunil 2014; Harrison and Sidebottom 2009)
Diagnostic psychiatric interview	2 (Yeung and Schwartz 1986; Yoshida et al. 1997)
Beck Depression Inventory-Fast Screen (BDI-FS) (Beck et al. 2000)	1 (Valentine et al. 2011)
Beck Depression Inventory-II (BDI-II) (Beck et al. 1996)	1 (Hung et al. 2012)
Depression Anxiety Stress Scales (DASS) (Lovibond and Lovibond 1995)	1 (Yelland et al. 2010)
Modified malaise inventory score (Sacker et al. 2006)	1 (Jayaweera and Quigley 2010)
Postpartum Depression Screening Scale (PDSS) (Beck and Gable 2002)	1 (Goyal et al. 2006)

#### Antenatal depression

Sixteen studies provided data for prevalence of antenatal elevated depression symptoms among a total of 5110 migrant women (Balestrieri et al. 2012; Diaz et al. 2007; Elo and Culhane 2010; Escribà-Agüir et al. 2013; Fleuriet and Sunil 2014; Fortner et al. 2011; Harrison and Sidebottom 2009; Heilemann et al. 2004; Luecken et al. 2013; McDonald et al. 2013; Miszkurka et al. 2010; Peer et al. 2013; Ratcliff et al. 2015; Rudman et al. 2008; Tsao et al. 2014; Zelkowitz et al. 2008). Random effects meta-analysis was used to calculate a pooled estimate of the prevalence, and estimate the level of heterogeneity between the studies. The *I* ^2^ value was over 98%, suggesting very high levels of heterogeneity between studies, and so the pooled estimate is not reported (analyses available on request). Heterogeneity was explored using sensitivity analyses that excluded studies with high risk of selection bias, and stratifying analyses by depression measurement and by study country; however, none of these factors influenced the *I* ^2^ value. The median prevalence of antenatal elevated depression symptoms among migrant women was 28%, with estimates ranging from 12% (Luecken et al. 2013) to 45% (Heilemann et al. 2004).

#### Postnatal depression

Thirty-three studies provided data for prevalence of postnatal elevated depression symptoms among a total of 15,153 migrant women (Abbott and Williams 2006; Balbierz et al. 2014; Ballantyne et al. 2013; Bandyopadhyay et al. 2010; Bjerke et al. 2008; Chen et al. 2012, 2013; Chien et al. 2012; Diaz et al. 2007; Fisch et al. 1997; Gagnon et al. 2013; Ganann et al. 2012; Glasser et al. 2000; Heilemann et al. 2004; Huang and Mathers 2008; Huang et al. 2007; Hung et al. 2012; Jayaweera and Quigley 2010; McDonald et al. 2013; Mechakra-Tahiri et al. 2007; Milgrom et al. 2008; Minde et al. 2001; Rudman et al. 2008; Shafiei et al. 2015; Small et al. 2003; Stewart et al. 2008, 2012; Sword et al. 2006; Tsao et al. 2014; Van Lieshout et al. 2011; Yelland et al. 2010; Yoshida et al. 1997; Zelkowitz et al. 2008). Heterogeneity between studies was very high with an *I* ^2^ value over 97%, so the pooled estimate from the random effects meta-analysis is not reported (analyses available on request). Again sensitivity analyses (removing studies with high risk of selection bias and stratifying by measurement and study country) had almost no effect on the level of heterogeneity. The median prevalence of postnatal elevated depression symptoms among migrant women was 19%, with estimates ranging from <1% (Hung et al. 2012) to 59% (Ballantyne et al. 2013).

### Risk factors for mental disorders in the perinatal period

Factors significantly associated with antenatal and postnatal elevated depression symptoms in studies included in the review are listed in Table 2 (see Table 2 for references to risk factors discussed).

**Table 2:** Risk factors for depression identified by included studies

Antenatal depression	Postnatal depression
■ Lack of social support (Miszkurka et al. 2010; Peer et al. 2013)) ■ Marital strain/lack marital support (Miszkurka et al. 2010; Ratcliff et al. 2015) ■ Time in host country ○ Increased length of residence in host country (Fleuriet and Sunil 2014) ○ Short duration in host country (Ratcliff et al. 2015) ○ Exposure to US in childhood (Heilemann et al. 2004) ■ Socioeconomic difficulty ○ Lack of money for basic needs (Miszkurka et al. 2010) ○ Housing difficulties (Ratcliff et al. 2015) ■ Stress/mental health ○ Life event distress (Miszkurka et al. 2010) ○ More perceived stress (Peer et al. 2013) ○ More somatic symptoms (Peer et al. 2013) ■ More acculturation (Fortner et al. 2011) ■ Not working or attending school in pregnancy (Peer et al. 2013) ■ Precarious legal status (Ratcliff et al. 2015)	■ Lack of social support (Chen et al. 2013; Chien et al. 2012; Small et al. 2003; Stewart et al. 2008; Tsao et al. 2014) ■ Ethnicity/country of birth (Bandyopadhyay et al. 2010; Jayaweera and Quigley 2010; Stewart et al. 2008) ■ Refugee or asylum-seeker status (Gagnon et al. 2013; Stewart et al. 2008) ■ Marital disharmony (Glasser et al. 2000; Zelkowitz et al. 2008) ■ Less proficient English (Bandyopadhyay et al. 2010; Small et al. 2003; Yelland et al. 2010) ■ Higher score life events (Bjerke et al. 2008; Yoshida et al. 1997) ■ Time in host country ○ Increased length of residence in host country (Jayaweera and Quigley 2010) ○ Short duration in host country (Small et al. 2003) ○ Exposure to US in childhood (Heilemann et al. 2004) ■Socioeconomic difficulty ○ Perceived family income insufficiency (Chien et al. 2012) ○ Higher levels of difficult life circumstances (Tsao et al. 2014) ■ Stress/mental health ○ History of depression (Bjerke et al. 2008) ○ History of emotional problems (Glasser et al. 2000) ○ Emotional distress following birth (Huang and Mathers 2008) ○ More childcare stress (Tsao et al. 2014) ○ More psychological distress (Tsao et al. 2014) ○ Antenatal depression (Zelkowitz et al. 2008) ○ More somatic symptoms (Zelkowitz et al. 2008) ○ High perinatal anxiety (Zelkowitz et al. 2008) ○ Pre-migration stress (Zelkowitz et al. 2008) ■ Age: >30 years (Bjerke et al. 2008), <19 (Glasser et al. 2000) ■ Not ‘doing-the-month’ (Chen et al. 2012) ■ Low domestic decision-making power (Chien et al. 2012) ■ Physical ill health (Small et al. 2003) ■ Marriage as reason for migration (Small et al. 2003) ■ Abused (Stewart et al. 2012) ■ Low birth weight infant (Tsao et al. 2014) ■ Obstetric difficulty (Yoshida et al. 1997)

Lower social support and lower marital support were consistently found as risk factors for depression symptoms in a number of studies. Variables related to socioeconomic difficulty and problems with stress or mental health were also consistently found to be risk factors for depressive symptoms. Less proficient host country language was found to be a consistent risk factor for postnatal depression in the studies that investigated it. Only two studies investigated refugee or asylum-seeker status, but both found increased risk of depressive symptoms among these groups compared to non-refugee or asylum-seeker migrants. There were contradictory findings for length of time in host country, with some studies finding an increased risk of depression associated with shorter duration in the host country, and others with longer duration in the host country. Due to differences in measures and lack of availability of data for the risk factor analyses from individual studies, we were unable to pool estimates for the strength of association with the risk factors identified.

### Risk of depression associated with migrant status

#### Antenatal depression

**Fig. 2:**
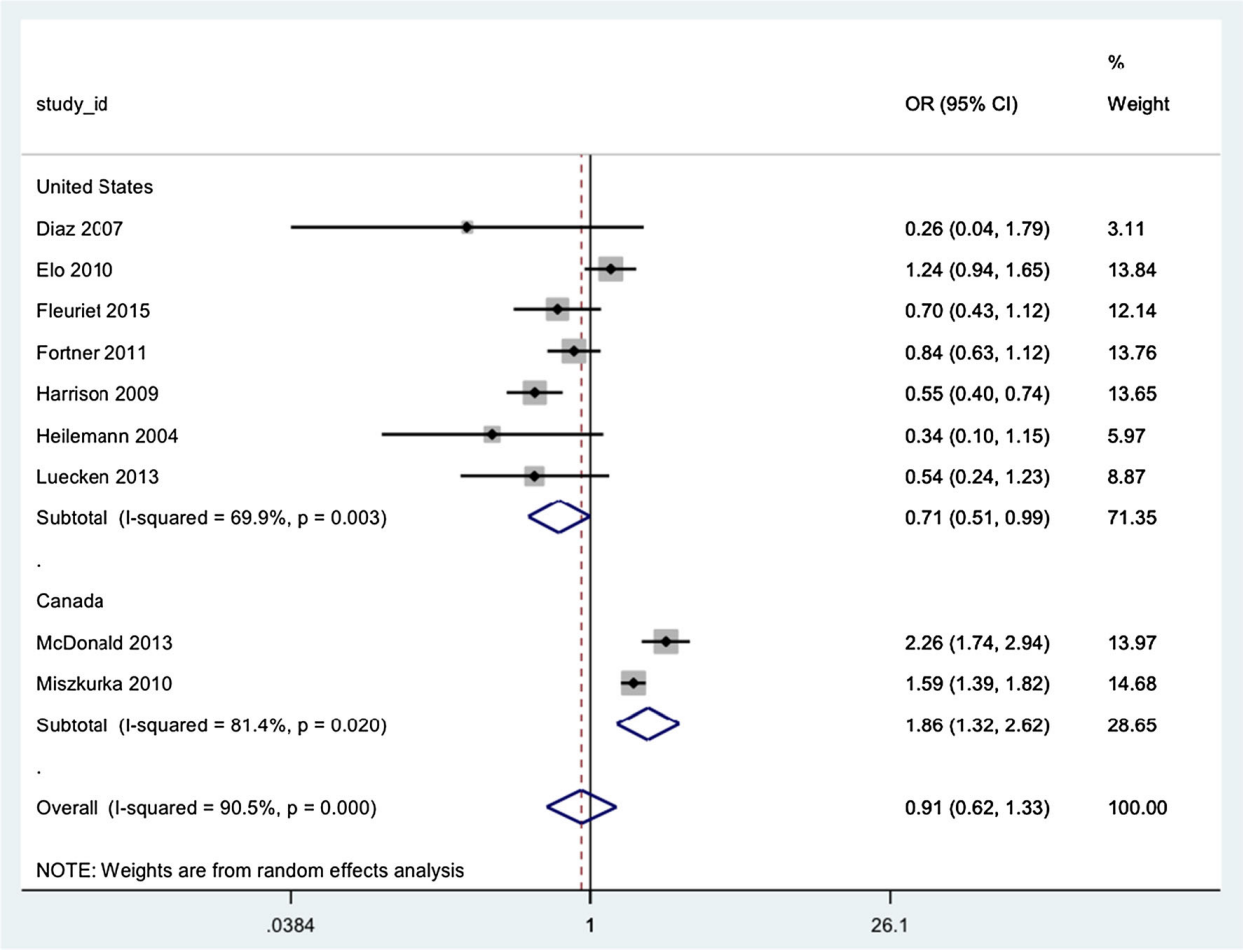
Forest plot of odds ratios for antenatal elevated depression symptoms associated with migrant status using random effects meta-analysis, stratified by study country

Twelve studies that provided data for the odds ratio associated with migrant status and antenatal elevated depression symptoms were included in a random effects meta-analysis (Balestrieri et al. 2012; Diaz et al. 2007; Elo and Culhane 2010; Escribà-Agüir et al. 2013; Fleuriet and Sunil 2014; Fortner et al. 2011; Harrison and Sidebottom 2009; Heilemann et al. 2004; Luecken et al. 2013; McDonald et al. 2013; Miszkurka et al. 2010; Rudman et al. 2008), including a total of 4776 migrant women and 15,773 non-migrant women (see Fig. 2). The *I* ^2^ measure of heterogeneity was 91.7%. A number of sensitivity analyses were then undertaken to explore the heterogeneity. Excluding the five studies with high risk of selection bias and stratifying by measure of depression (CES-D, EPDS and PHQ-9) had little effect on the level of heterogeneity. Finally, the meta-analysis was conducted stratifying for study country. Only two countries, the USA and Canada, had more than one study. The US meta-analysis included seven studies and the *I* ^2^ decreased to 69.9%. The pooled estimate for the OR was 0.71 (0.51, 0.99) suggesting that migrant women may have a decreased risk of antenatal elevated depression symptoms compared with US-born counterparts. The heterogeneity in the Canadian studies was still high (*I* ^2^ = 81.4%), but the pooled OR of 1.86 (1.32, 2.62) suggests that there may be an increased risk of antenatal elevated depression symptoms for migrant women in Canada.

The variation in the study populations included from the USA and Canada may explain the difference in results. Six out of the seven US studies only included women of one ethnicity (e.g. self-identifying as Mexican or Mexican American). The remaining US study included a population that was ‘predominantly women of colour’. All of the US study populations were comprised of low-income women. Therefore, the US studies controlled for the effects of income and minority ethnicity. In comparison, the Canadian studies included a range of ethnicities and income levels in their samples.

#### Postnatal depression

**Fig. 3:**
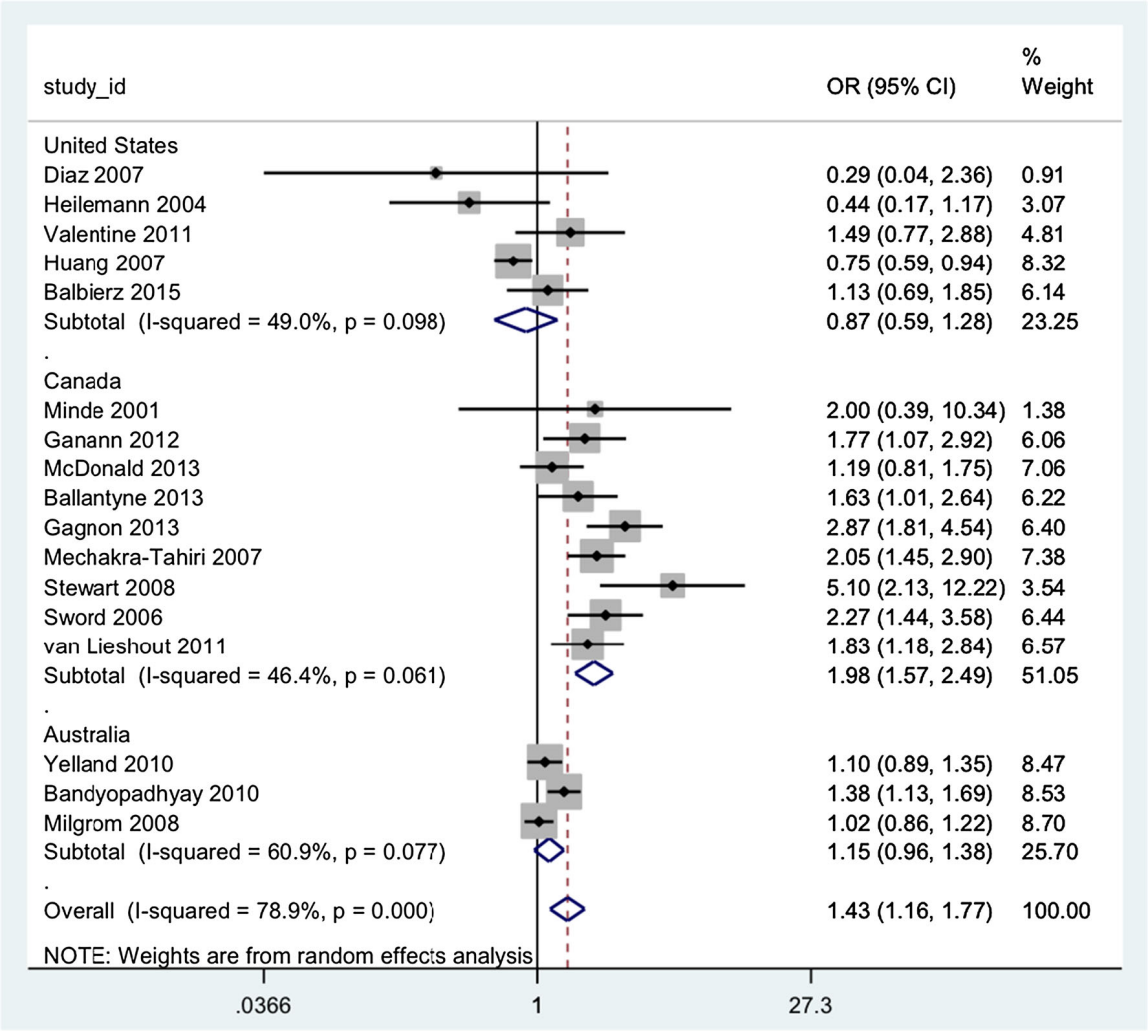
Forest plot of odds ratios for postnatal elevated depression symptoms associated with migrant status using random effects meta-analysis, stratified by study country

Twenty-four studies that provided data for the OR associated with migrant status and postnatal elevated depression symptoms were included in a random effects meta-analysis (Abbott and Williams 2006; Balbierz et al. 2014; Ballantyne et al. 2013; Bandyopadhyay et al. 2010; Chien et al. 2012; Choi et al. 2012; Diaz et al. 2007; Fisch et al. 1997; Gagnon et al. 2013; Ganann et al. 2012; Glasser et al. 2000; Heilemann et al. 2004; Huang et al. 2007; Jayaweera and Quigley 2010; McDonald et al. 2013; Mechakra-Tahiri et al. 2007; Milgrom et al. 2008; Minde et al. 2001; Rudman et al. 2008; Stewart et al. 2008; Sword et al. 2006; Valentine et al. 2011; Van Lieshout et al. 2011; Yelland et al. 2010), including a total of 12,741 migrant women and 53,259 non-migrant women (see Fig. 3). The pooled estimate for the OR was 1.56 (1.31, 1.86), suggesting an overall increased risk of postnatal elevated depression symptoms associated with migrant status. The *I* ^2^ measure of heterogeneity was 80.7%. Again, excluding studies with high risk of bias and stratifying by depression measure had no effect on heterogeneity. However, stratifying by study country explained some of the heterogeneity. Three countries contributed three or more studies: Canada (nine studies), the USA (five studies) and Australia (three studies). Pooled estimates suggest that there is no association between migration and postnatal elevated depression symptoms in the USA (OR = 0.87; 95% CIs 0.58, 1.28; *I* ^2^ 49.0%) or Australia (OR = 1.15; 95% CIs 0.96, 1.38; *I* ^2^ 60.9%); however, there is an increased risk of postnatal elevated depression symptoms associated with migrant status in Canada (OR = 1.98; 95% CIs 1.57, 2.49; *I* ^2^ 46.4%).

As with the antenatal depression studies, most of the studies from the USA were conducted in low-income minority populations, thus controlling for effects of ethnicity and socioeconomic status. The one nationally representative study from the USA compared migrant and non-migrant risk for depression by ethnicity and found that for non-Hispanic white women there was no difference by nativity. Foreign-born Black and Hispanic mothers had lower prevalence than their native-born counterparts, but foreign-born Asian mothers had higher prevalence than their native-born counterparts.

In contrast, the Canadian studies did not limit samples to particular ethnicities. However, two of the studies explicitly included asylum-seeking and refugee women, and three of the studies limited the migrant group to those who had been living in Canada for less than 5 or 10 years. One study that disaggregated migrant groups by ethnicity found no increase of depressive symptoms among migrant women from majority ethnic backgrounds, but an increase for minority ethnic backgrounds.

Of the Australian studies, the only one that found an increased risk associated with migrant status limited the migrant group to those from a non-English-speaking background (NESB). One of the other studies that found no overall significant odds difference also found increased depression and anxiety symptoms for migrants from NESB backgrounds.

### Other disorders

Only three studies looked at disorders other than depression, thus it was not possible to pool estimates for each of these disorders, instead their results are presented individually.

#### Posttraumatic stress disorder

One study looked at the prevalence of PTSD among postpartum migrant women who had been living in the host country, Canada, for less than 5 years (Gagnon et al. 2013). PTSD was measured by reporting ‘a lot’ or ‘extremely’ to one or more trauma symptom(s) in the last week on the Harvard Trauma Questionnaire. The migrant group was split into three statuses: asylum-seekers, refugees and immigrants (women with non-refugee histories). There were also Canadian-born women included in the study (non-aboriginal, living in Canada majority of their lives), although there was no prevalence of PTSD reported for this group. Asylum-seeking women had the highest prevalence of PTSD with 48% above the cut-off, followed by refugees (34%) and immigrants (15%).

#### Anxiety

One Australian study measured anxiety at 6 months postpartum in addition to depression, using the Depression Anxiety Stress Scales (DASS) (Yelland et al. 2010). They looked at levels of comorbidity of women experiencing anxiety and depression as well. The migrant women were women born outside of Australia, but they were divided into those from English-speaking and NESB. They found that women born overseas with NESB were at increased risk of both anxiety and comorbid anxiety and depression, but that there was no difference in risk of disorder for women from English-speaking backgrounds compared to the Australian-born women.

#### Any DSM-III diagnosis

One small study looking at Chinese migrant women living in New York investigated any DSM-III diagnosis during pregnancy, using a psychiatric interview (Yeung and Schwartz 1986). Twenty-eight of 124 patients (23%) received DSM-III diagnoses. Recent immigrants (resided in the USA for less than 1 year) were over-represented in the group of women given DSM-III diagnoses.

## Discussion

This review found that depression is common among pregnant and postpartum migrant women, although there was no evidence for an overall increased risk of depression among migrant women when compared to non-migrant women. However, this must be interpreted with consideration of the very different populations included in the migrant and non-migrant groups between studies. The review found that only three studies investigated a mental disorder other than depression among perinatal migrant women, and in these studies, the migrant women were at increased risk of anxiety symptoms, and asylum-seeking and refugee women had high prevalence of PTSD symptoms.

### Prevalence and risk factors

Antenatal and postnatal depression appear to be at least as common among migrant women as in the general population (Gavin et al. 2005). Most of the risk factors identified are the same as found in population-based samples, such as lack of social support, marital disharmony, socioeconomic difficulty, minority ethnicity and stress (Howard et al. 2014). However, there are also risk factors, such as time in host country, lack of proficiency in host country language and precarious legal status that are unique to migrant women. With the increased likelihood of exposure to risk factors among migrant women, it was expected that we would find an increased risk of antenatal and postnatal depression among migrant women when compared to non-migrant women.

### Comparison with non-migrant women

This review found no evidence for an overall increased risk of antenatal or postnatal depression among migrant women compared to non-migrant women. However, when stratified by study country, migrant women in Canada were at increased risk of antenatal and postnatal depression compared to their native-born counterparts, whereas migrant women in America and Australia were not. This may be explained by looking at the differences between the migrant and non-migrant populations included in the studies from these countries, for example, in relation to race or ethnicity.

Minority ethnicity is a risk factor for mental disorders and has been shown to be a risk factor for perinatal depression. Most of the US studies controlled for ethnicity inadvertently by only including minority ethnicities in samples (e.g. populations of low-income women who self-identified as Latina); this may explain the lack of increased risk for migrant women. The only nationally representative cohort from the USA looked at odds of postnatal depression associated with being foreign-born within each ethnicity (Huang et al. 2007). For non-Hispanic whites, there was no difference. For Black and Hispanic women, foreign-born mothers had lower prevalence of depressive symptoms than native-born counterparts. Foreign-born Asian mothers had higher prevalence than native-born counterparts. Furthermore, one of the Canadian studies found that only migrant women belonging to minority groups had increased depressive symptoms compared to majority ethnicity migrants and Canadian-born (Mechakra-Tahiri et al. 2007). Thus, it appears that race, ethnicity and migration need to be considered in conjunction as intersecting risk factors for mental disorders.

Experiences of stressors associated with being a minority, such as day to day experiences of discrimination, have been suggested to have a cumulative detrimental effect on health (Kessler et al. 1999). This may help to explain the deterioration of migrant health over time in the host country despite increasing socioeconomic status (De Maio and Kemp 2010; Viruell-Fuentes et al. 2012). One large cohort study in the UK included in the review found this effect for maternal mental and physical health, with an initial healthy effect that decreased over time (Jayaweera and Quigley 2010). This may explain the decreased risk of perinatal depression among US migrants when compared to their native-born ethnic counterparts, as being born outside of the USA has given them less cumulative exposure to the stresses of being a minority. In the Canadian samples, the migrant groups were being compared to a native-born population with a majority ethnicity; thus, the effects of minority stressors in the native-born population would not be observed and the risk differential increased.

Examining differences in the migrant and non-migrant groups between different study countries in terms of exposure to risk factors, such as poverty, social support, language proficiency and legal status, may provide further possible explanations for the difference in risk observed.

Many migrants are likely to experience poverty in the host country, which is a key social determinant for both mental and physical health (Jayaweera 2011). Many of the studies, particularly those conducted in the USA, were looking at low-income populations, and so the SES difference between migrant and non-migrant would be less than at a population level, again reducing the risk difference.

A low level of social support is one of the main risk factors for depression in the perinatal period for migrant women (Collins et al. 2011a; Miszkurka et al. 2012a). Levels of social support are likely to be lower among migrant women due to separation from social networks through the migration process and social isolation in the host country. It is possible that in the studies from the USA, the majority of which were conducted in populations with high proportions of migrants and non-migrants from the same cultural background, the differential social isolation between foreign and native-born women is less.

A lack of proficiency in the host country language is another factor associated with mental disorders among migrant women (Bandyopadhyay et al. 2010; Small et al. 2003; Yelland et al. 2010). One Australian study looking at postnatal depression and anxiety found that migrants from an English-speaking background were at no increased risk, whereas migrant women from a non-English-speaking background were at an increased risk (Yelland et al. 2010). However, many studies included in the review excluded women who were unable to speak the host country language, thus reducing the risk difference between migrant and non-migrant.

Refugee and asylum-seeker women are found to have higher levels of mental disorders, not only because they are more likely to have experienced traumatic life events (Hollifield et al. 2002), but also because insecure migrant status itself and the asylum process can cause great anxiety (Coffey et al. 2010; Silove et al. 1997). The few studies that looked at refugee and asylum-seeking women separately did find evidence for an increased risk of depression and high levels of PTSD symptoms (Gagnon et al. 2013; Stewart et al. 2008). However, most studies did not include these hard-to-reach vulnerable migrants, and therefore, the risk difference between migrant and non-migrant would again be reduced. Whereas the stressors of being a minority may cumulate over time, it is also possible that for women fleeing violence or with insecure migration status, shorter length of time in host country could be a risk factor for mental disorders. This may help to explain discrepancies in findings around length of time in host country and depression. The effect of time in the host country may differ depending on the reason for migration: with a recency effect for those fleeing trauma and violence and a cumulative effect of minority-related stressors.

### Strengths and limitations

This is the first comprehensive review of the literature on migration and perinatal mental disorders including all migrant populations. We were able to include large numbers of studies in this review, despite the small number of studies investigating the effect of migrant status on perinatal mental disorders, through extensive contact of authors for data. By looking in detail at the sources of heterogeneity between studies, we have been able to point to potential reasons for the contradictory findings in this area, which extends beyond the scope of the review to research on mental health of migrant populations more generally.

There were no studies conducted in low- and middle-income countries, which reduces generalizability, especially as the characteristics of migrant populations vary so much across different countries. The inclusion of only English language papers due to limited resources also reduces generalizability. The broad inclusion of different studies and different populations meant that heterogeneity was very high for the prevalence estimates; however, this breadth of inclusion was also a strength of the review as it allowed for comparisons between these different populations.

The lack of high-quality research in this area is the greatest limitation of the review. There were very few studies with a low risk of selection bias. Many studies excluded women who did not speak the study country language, specified inclusion by ethnic group or SES and likely excluded migrants with insecure migration status, all of which would have biased the main association of interest.

There were very few studies with a low risk of measurement bias, as the vast majority of studies used a screening tool to measure mental disorder rather than a diagnostic tool. When making cross-cultural comparisons of risk of disorder, it is particularly important that the tools are valid across different translations. The studies included in this review mainly used the EPDS to measure depression, which has been found to show huge variation in sensitivity and specificity across cultural groups in a systematic review of different translations (Gibson et al. 2009). Other studies used standard screening measures such as the CES-D or PHQ-9, which may over-diagnose women in the perinatal period (Harris et al. 1989). In comparing women from different cultures, it is important that the measures have high reliability. Despite this, the sensitivity analyses suggested that at least the different tools of measurement were not accounting for much of the variance between studies.

### Implications

Depression is common in the perinatal period among migrant women, and there are women who are likely to be particularly vulnerable on the basis of migrant status in combination with social isolation, minority ethnicity, low SES, insecure legal status, poor language proficiency and many other factors. However, there is a paucity of research addressing the heterogeneity of migrant populations and investigating these intersecting factors, and there is almost no research looking at disorders other than depression in the perinatal period. We need research that uses diagnostic measures of mental disorder to be able to estimate valid differences in risk across different cultural groups. Studies need to attempt to recruit hard-to-reach groups of migrants such as those with insecure legal status and those who do not speak the study country language, as these groups are likely to be at high risk of mental disorder but also are likely to be missed by health services. With the number of displaced persons at its historical highpoint in the current refugee crisis, research into the health of migrant populations has never been more important. In health services, this will work towards better identification of women at risk, and being able to offer interventions that meet the specific needs of increasingly heterogeneous populations. The broader social implication is the urgent need to address the stressors that migrant women face, such as discrimination, poverty and social isolation, in a global environment that is increasingly hostile towards migrants.

## Electronic supplementary material

ESM 1 (PDF 197 kb)

ESM 2 (PDF 422 kb)

## References

[CR1] Abbott MW, Williams MM (2006). Postnatal depressive symptoms among Pacific mothers in Auckland: prevalence and risk factors. Aust N Z J Psychiatry.

[CR2] Balbierz A, Bodnar-Deren S, Wang JJ, Howell EA (2014) Maternal depressive symptoms and parenting practices 3-months postpartum. Maternal and Child Health Journal Nov:no pagination specified. doi:10.1007/s10995-014-1625-6 10.1007/s10995-014-1625-6 PMC4422772 25374288

[CR3] Balestrieri M (2012). Determinants of ante-partum depression: a multicenter study.[Erratum appears in Soc Psychiatry Psychiatr Epidemiol. 2013 Mar; 48(3):513 note: multiple author names corrected]. Social Psychiatry & Psychiatric Epidemiology.

[CR4] Ballantyne M, Benzies KM, Trute B (2013). Depressive symptoms among immigrant and Canadian born mothers of preterm infants at neonatal intensive care discharge: a cross sectional study. BMC Pregnancy and Childbirth.

[CR5] Bandyopadhyay M, Small R, Watson LF, Brown S (2010). Life with a new baby: how do immigrant and Australian-born women’s experiences compare?. Australian & New Zealand Journal of Public Health.

[CR6] Bauer A, Parsonage M, Knapp M, Iemmi V, Adelaja B, Hogg S (2014). The costs of perinatal mental health problems.

[CR7] Beck A, Steer R, Brown G (1996) Manual for the Beck Depression Inventory-II. Psychological Corporation, San Antonio

[CR8] Beck A, Steer R, Brown G (2000) BDI-Fast Screen for medical patients: manual. The Psychological Corporation., San Antonio

[CR9] Beck C, Gable R (2002) The postpartum depression screening scale manual. Western Psychological Association, Los Angeles

[CR10] Bjerke SEY, Vangen S, Nordhagen R, Ytterdahl T, Magnus P, Stray-Pedersen B (2008). Postpartum depression among Pakistani women in Norway: prevalence and risk factors. J Matern Fetal Neonatal Med.

[CR11] Chen T-L, Tai C-J, Wu T-W, Chiang C-P, Chien L-Y (2012). Postpartum cultural practices are negatively associated with depressive symptoms among Chinese and Vietnamese immigrant mothers married to Taiwanese men. Women & Health.

[CR12] Chen H-H, Hwang F-M, Tai C-J, Chien L-Y (2013). The interrelationships among acculturation, social support, and postpartum depression symptoms among marriage-based immigrant women in Taiwan: a cohort study. Journal of immigrant and minority health/Center for Minority Public Health.

[CR13] Chien L-Y, Tai C-J, Yeh M-C (2012). Domestic decision-making power, social support, and postpartum depression symptoms among immigrant and native women in Taiwan. Nurs Res.

[CR14] Choi SY, Kim EJ, Ryu E, Chang KO, Park MN (2012). Postpartum depression and parental self-efficacy: a comparison of native Korean and Vietnamese immigrant mothers in Korea … [corrected] [published erratum appears in J TRANSCULT NURS 2012; 23(3):329]. J Transcult Nurs.

[CR15] Coffey GJ, Kaplan I, Sampson RC, Tucci MM (2010). The meaning and mental health consequences of long-term immigration detention for people seeking asylum. Soc Sci Med.

[CR16] Collins CH, Zimmerman C, Howard LM (2011). Refugee, asylum seeker, immigrant women and postnatal depression: rates and risk factors. Arch Womens Ment Health.

[CR17] Collins CH, Zimmerman C, Howard LM (2011). Refugee, asylum seeker, immigrant women and postnatal depression: rates and risk factors. Archives of Women’s Mental Health.

[CR18] Connelly CD, Hazen AL, Baker-Ericzén MJ, Landsverk J, Horwitz SM (2013). Is screening for depression in the perinatal period enough? The co-occurrence of depression, substance abuse, and intimate partner violence in culturally diverse pregnant women. J Women’s Health.

[CR19] Cox JL, Holden JM, Sagovsky R (1987) Detection of postnatal depression. Development of the 10-item Edinburgh Postnatal Depression Scale Br J Psychiatry 150:782-786 10.1192/bjp.150.6.782 3651732

[CR20] Critical Appraisal Skills Programme (CASP) (2014) CASP checklists. (http://media.wix.com/ugd/dded87_63fb65dd4e0548e2bfd0a982295f839e.pdf; http://media.wix.com/ugd/dded87_e37a4ab637fe46a0869f9f977dacf134.pdf). CASP, Oxford

[CR21] Davila M, McFall SL, Cheng D (2009). Acculturation and depressive symptoms among pregnant and postpartum. Latinas Maternal & Child Health Journal.

[CR22] De Maio FG, Kemp E (2010). The deterioration of health status among immigrants to Canada. Glob Public Health.

[CR23] Dennis CL, Janssen PA, Singer J (2004). Identifying women at-risk for postpartum depression in the immediate postpartum period. Acta Psychiatr Scand.

[CR24] Diaz MA, Le HN, Cooper BA, Munoz RF (2007). Interpersonal factors and perinatal depressive symptomatology in a low-income Latina sample. Cultur Divers Ethnic Minor Psychol.

[CR25] Downs SH, Black N (1998). The feasibility of creating a checklist for the assessment of the methodological quality both of randomised and non-randomised studies of health care interventions. J Epidemiol Community Health.

[CR26] Eastwood JG, Phung H, Barnett B (2011). Postnatal depression and socio-demographic risk: factors associated with Edinburgh Depression Scale scores in a metropolitan area of New South Wales, Australia. Aust N Z J Psychiatry.

[CR27] Elo IT, Culhane JF (2010). Variations in health and health behaviors by nativity among pregnant Black women in Philadelphia. Am J Public Health.

[CR28] Escribà-Agüir V, Royo-Marqués M, Artazcoz L, Romito P, Ruiz-Pérez I (2013). Longitudinal study of depression and health status in pregnant women: incidence, course and predictive factors. Eur Arch Psychiatry Clin Neurosci.

[CR29] Fellmeth G, Fazel M, Plugge E (2016). Migration and perinatal mental health in women from low- and middle-income countries: a systematic review and meta-analysis. BJOG.

[CR30] Fisch RZ, Tadmor OP, Dankner R, Diamant YZ (1997). Postnatal depression: a prospective study of its prevalence, incidence and psychosocial determinants in an Israeli sample. Journal of Obstetrics & Gynaecology Research.

[CR31] Fleuriet KJ, Sunil TS (2014). Perceived social stress, pregnancy-related anxiety, depression and subjective social status among pregnant Mexican and Mexican American women in south Texas. Journal of Health Care for the Poor & Underserved.

[CR32] Fortner R, Pekow P, Dole N, Markenson G, Chasan-Taber L (2011). Risk factors for prenatal depressive symptoms among Hispanic women. Maternal & Child Health Journal.

[CR33] Gagnon AJ (2009). Migration to western industrialised countries and perinatal health: a systematic review. Soc Sci Med.

[CR34] Gagnon AJ (2013). International migration to Canada: the post-birth health of mothers and infants by immigration class. Soc Sci Med.

[CR35] Ganann R, Sword W, Black M, Carpio B (2012). Influence of maternal birthplace on postpartum health and health services use. Journal of Immigrant & Minority Health.

[CR36] Gavin NI, Gaynes BN, Lohr KN, Meltzer-Brody S, Gartlehner G, Swinson T (2005). Perinatal depression: a systematic review of prevalence and incidence. Obstet Gynecol.

[CR37] Gibson J, McKenzie-McHarg K, Shakespeare J, Price J, Gray R (2009). A systematic review of studies validating the Edinburgh Postnatal Depression Scale in antepartum and postpartum women. Acta Psychiatr Scand.

[CR38] Glasser S, Barell V, Boyko V, al. e (2000) Postpartum depression in an Israeli cohort: demographic, psychosocial and medical risk factors. J Psychosom Obstet Gynecol 21:99–108 10.3109/01674820009075615 10994182

[CR39] Goyal D, Murphy SO, Cohen J (2006). Immigrant Asian Indian women and postpartum depression. JOGNN-Journal of Obstetric, Gynecologic, & Neonatal Nursing.

[CR40] Harris B, Huckle P, Thomas R, Johns S, Fung H (1989). The use of rating scales to identify post-natal depression. Br J Psychiatry.

[CR41] Harrison PA, Sidebottom AC (2009). Alcohol and drug use before and during pregnancy: an examination of use patterns and predictors of cessation. Maternal & Child Health Journal.

[CR42] Heilemann MV, Frutos L, Lee KA, Kury FS (2004). Protective strength factors, resources, and risks in relation to depressive symptoms among childbearing women of Mexican descent. Health Care for Women International.

[CR43] Hollifield M (2002). Measuring trauma and health status in refugees. JAMA.

[CR44] Howard LM, Molyneaux E, Dennis C-L, Rochat T, Stein A, Milgrom J (2014). Non-psychotic mental disorders in the perinatal period. Lancet.

[CR45] Huang YC, Mathers NJ (2008). Postnatal depression and the experience of South Asian marriage migrant women in Taiwan: survey and semi-structured interview study. Int J Nurs Stud.

[CR46] Huang ZJ, Wong FY, Ronzio CR, al. e (2007) Depressive symptomatology and mental health help-seeking patterns of U.S.- and foreign-born mothers. Matern Child Health J 11:257–267 10.1007/s10995-006-0168-x 17171544

[CR47] Hung C-H, Wang H-H, Chang S-H, Jian S-Y, Yang Y-M (2012). The health status of postpartum immigrant women in Taiwan. J Clin Nurs.

[CR48] Jayaweera H (2011). Health of migrants in the UK: what do we know?.

[CR49] Jayaweera H, Quigley M (2010). Health status, health behaviour and healthcare use among migrants in the UK: evidence from mothers in the Millennium Cohort Study. Soc Sci Med.

[CR50] Kessler RC, Mickelson KD, Williams DR (1999). The prevalence, distribution, and mental health correlates of perceived discrimination in the United States. J Health Soc Behav.

[CR51] Lanes A, Kuk JL, Tamim H (2011). Prevalence and characteristics of postpartum depression symptomatology among Canadian women: a cross-sectional study. BMC Public Health.

[CR52] Lovibond S, Lovibond P (1995) Manual for the Depression Anxiety Stress Scales. 2 edition. Psychology Foundation, Sydney

[CR53] Luecken LJ, Lin B, Coburn SS, MacKinnon DP, Gonzales NA, Crnic KA (2013). Prenatal stress, partner support, and infant cortisol reactivity in low-income Mexican American families. Psychoneuroendocrinology.

[CR54] MacLehose RR, Reeves BC, Harvey IM, Sheldon TA, Russell IT, Black AM (2000). A systematic review of comparisons of effect sizes derived from randomised and non-randomised studies. Health Technol Assess.

[CR55] McDonald SW (2013). The All Our Babies pregnancy cohort: design, methods, and participant characteristics. BMC Pregnancy & Childbirth.

[CR56] Mechakra-Tahiri S, Zunzunegui MV, Sguin L (2007). Self-related health and postnatal depressive symptoms among immigrant mothers in Québec. Women & Health.

[CR57] Milgrom J (2008). Antenatal risk factors for postnatal depression: a large prospective study. J Affect Disord.

[CR58] Minde K, Tidmarsh L, Hughes S (2001). Nurses’ and physicians’ assessment of mother-infant mental health at the first postnatal visits. Journal of the American Academy of Child & Adolescent Psychiatry.

[CR59] Miszkurka M, Goulet L, Zunzunegui MV (2010). Contributions of immigration to depressive symptoms among pregnant women in Canada. Can J Public Health.

[CR60] Miszkurka M, Goulet L, Zunzunegui MV (2012). Antenatal depressive symptoms among Canadian-born and immigrant women in Quebec: differential exposure and vulnerability to contextual risk factors. Social Psychiatry & Psychiatric Epidemiology.

[CR61] Miszkurka M, Goulet L, Zunzunegui MV (2012). Antenatal depressive symptoms among Canadian-born and immigrant women in Quebec: differential exposure and vulnerability to contextual risk factors. Soc Psychiatry Psychiatr Epidemiol.

[CR62] Peer M, Soares CN, Levitan RD, Streiner DL, Steiner M (2013). Antenatal depression in a multi-ethnic, community sample of Canadian immigrants: psychosocial correlates and hypothalamic-pituitary-adrenal axis function. Canadian Journal of Psychiatry-Revue Canadienne de Psychiatrie.

[CR63] Radloff LS (1977) The CES-D Scale: A Self-Report Depression Scale for Research in the General Population Applied Psychological Measurement 1:385-401 doi:10.1177/014662167700100306

[CR64] Ratcliff BG, Sharapova A, Suardi F, Borel F (2015). Factors associated with antenatal depression and obstetric complications in immigrant women in Geneva. Midwifery.

[CR65] Rechel B, Mladovsky P, Ingleby D, Mackenbach JP, McKee M (2013). Migration and health in an increasingly diverse Europe. Lancet.

[CR66] Rudman A, El-Khouri B, Waldenstrom U (2008). Evaluating multi-dimensional aspects of postnatal hospital care. Midwifery.

[CR67] Sacker A, Quigley MA, Kelly YJ (2006) Breastfeeding and developmental delay: findings from the millennium cohort study Pediatrics 118:e682-689 doi:10.1542/peds.2005-3141 10.1542/peds.2005-3141 16950960

[CR68] Shafiei T, Small R, McLachlan H (2015). Immigrant Afghan women’s emotional well-being after birth and use of health services in Melbourne, Australia. Midwifery.

[CR69] Silove D, Sinnerbrink I, Field A, Manicavasagar V, Steel Z (1997). Anxiety, depression and PTSD in asylum-seekers: associations with pre-migration trauma and post-migration stressors. Br J Psychiatry.

[CR70] Small R, Lumley J, Yelland J (2003). Cross-cultural experiences of maternal depression: associations and contributing factors for Vietnamese, Turkish and Filipino immigrant women in Victoria, Australia. Ethnicity & Health.

[CR71] Stein A (2014). Effects of perinatal mental disorders on the fetus and child. Lancet.

[CR72] Stewart DE, Gagnon A, Saucier JF (2008). Postpartum depression symptoms in newcomers. The Canadian Journal of Psychiatry.

[CR73] Stewart DE, Gagnon AJ, Merry LA, Dennis C-L (2012). Risk factors and health profiles of recent migrant women who experienced violence associated with pregnancy. J Women’s Health.

[CR74] Sword W, Watt S, Krueger P (2006). Postpartum health, service needs, and access to care experiences of immigrant and Canadian-born women. J Obstet Gynecol Neonatal Nurs.

[CR75] Tran M, Phung H, Young L, Hopper U, Hillman K (2002). Patterns and characteristics of ethnic Australian women utilising ethno-specific maternal and child health services. Aust Health Rev: Publ Australian Hospital Assoc.

[CR76] Trevillion K, Oram S, Feder G, Howard LM (2012). Experiences of domestic violence and mental disorders: a systematic review and meta-analysis. PLoS One.

[CR77] Tsao Y, Creedy DK, Gamble J (2014). Emotional well-being of Vietnamese immigrant women during the transition to motherhood: a descriptive cohort study. Nurs Health Sci.

[CR78] United Nations (2013). Trends in international migration stock.

[CR79] Valentine JM, Rodriguez MA, Lapeyrouse LM, Zhang M (2011). Recent intimate partner violence as a prenatal predictor of maternal depression in the first year postpartum among Latinas. Archives of Women’s Mental Health.

[CR80] Van Lieshout RJ, Cleverley K, Jenkins JM, Georgiades K (2011). Assessing the measurement invariance of the Center for Epidemiologic Studies Depression Scale across immigrant and non-immigrant women in the postpartum period. Archives of Women’s Mental Health.

[CR81] Vesga-Lopez O, Blanco C, Keyes K, Olfson M, Grant BF, Hasin DS (2008). Psychiatric disorders in pregnant and postpartum women in the United States. Arch Gen Psychiatry.

[CR82] Viruell-Fuentes E, Miranda PY, Abdulrahim S (2012). More than culture: structural racism, intersectionality theory, and immigrant health. Soc Sci Med.

[CR83] Wells GA, Shea B, O’Connell D, Peterson J, Welch V, Losos M, Tugwell P (2009). The Newcastle-Ottawa Scale (NOS) for assessing the quality of nonrandomised studies in meta-analyses.

[CR84] Yelland J, Sutherland G, Brown SJ (2010). Postpartum anxiety, depression and social health: findings from a population-based survey of Australian women. BMC Public Health.

[CR85] Yeung WH, Schwartz MA (1986). Emotional disturbance in Chinese obstetrical patients: a pilot study. Gen Hosp Psychiatry.

[CR86] Yoshida K, Marks MN, Kibe N, Kumar R, Nakano H, Tashiro N (1997). Postnatal depression in Japanese women who have given birth in England. J Affect Disord.

[CR87] Zelkowitz P, Saucier JF, Wang T, Katofsky L, Valenzuela M, Westreich R (2008). Stability and change in depressive symptoms from pregnancy to two months postpartum in childbearing immigrant women. Archives of Women’s Mental Health.

