# Supplementary material for: Prevalence and risk of mental disorders in the perinatal period among migrant women: a systematic review and meta-analysis
Source: Arch Womens Ment Health. 2017 Apr 8;20(3):449–62. doi: 10.1007/s00737-017-0723-z (PMC5423996; doi:10.1007/s00737-017-0723-z)
Supplement: Supplementary file 1 — (PDF 197 kb) [file 737_2017_723_MOESM1_ESM.pdf]

**Full list of search terms for electronic databases**

Medline

1. exp Human migration/
2. exp Refugees/
3. refugee* or immigrant* or migrant*or immigration or migration or foreign* or new?comer*
4. asylum adj2 seek*
5. 1 or 2 or 3 or 4

6. exp Pregnancy/
7. exp peripartum period/ or exp postpartum period/ or exp pregnancy trimesters/ or exp pregnancy, multiple/
8. pregnan* or post?partum or post?natal or puerperal or ante?natal or pre?natal or ante?partum or peri?natal or birth* or trimester*
9. 6 or 7 or 8

10. exp Mental Disorders/
11. exp Mental Health/
12. Mentally Ill Persons/
13. (mental* or psych*) adj2 (problem* or difficult* or disorder* or ill* or health*)
14. Schiz* or psychosis or psychotic or bi?polar or depress* or dysthimi* or mania or manic or neuros?s or psychoneuros?s or obsessive or compulsive or ocd or anxiety or ptsd or post?traumatic or panic or phobia
15. (delusional or paranoi* or mood or affective or neurotic or stress or reactive or combat or somatoform or somati?ation or phobic or adjustment or dissociat*) adj2 disorder*
16. Personality adj2 disorder*
17. eat* adj2 disorder*
18. Bulimi* or anorexi*
19. Bing* adj eat*
20. Compulsive adj (eat* or vomit* or purg*)
21. 10 or 11 or 12 or 13 or 14 or 15 or 16 or 17 or 18 or 19 or 20

22. 5 and 9 and 23

PsycINFO

1. exp Human migration/
2. exp Immigration/
3. refugee* or immigrant* or migrant*or immigration or migration or foreign* or new?comer*
4. asylum adj2 seek*
5. 1 or 2 or 3 or 4

6. exp Pregnancy/
7. exp peripartum period/ or exp postnatal period/ or exp birth/
8. pregnan* or post?partum or post?natal or puerperal or ante?natal or pre?natal or ante?partum or peri?natal or birth* or trimester*
9. 6 or 7 or 8

10. exp Mental Disorders/
11. exp Mental Health/
12. exp Psychiatric patients/
13. (mental* or psych*) adj2 (problem* or difficult* or disorder* or ill* or health*)
14. Schiz* or psychosis or psychotic or bi?polar or depress* or dysthimi* or mania or manic or neuros?s or psychoneuros?s or obsessive or compulsive or ocd or anxiety or ptsd or post?traumatic or panic or phobia
15. (delusional or paranoi* or mood or affective or neurotic or stress or reactive or combat or somatoform or somati?ation or phobic or adjustment or dissociat*) adj2 disorder*
16. Personality adj2 disorder*
17. eat* adj2 disorder*
18. Bulimi* or anorexi*
19. Bing* adj eat*
20. Compulsive adj (eat* or vomit* or purg*)
21. 10 or 11 or 12 or 13 or 14 or 15 or 16 or 17 or 18 or 19 or 20

22. 5 and 9 and 23

EMBASE

1. exp migration/
2. exp migrant/
3. refugee* or immigrant* or migrant*or immigration or migration or foreign* or new?comer*
4. asylum adj2 seek*
5. 1 or 2 or 3 or 4

6. exp Pregnancy/
7. exp perinatal period/ or exp puerperium/ or exp birth/
8. pregnan* or post?partum or post?natal or puerperal or ante?natal or pre?natal or ante?partum or peri?natal or birth* or trimester*
9. 6 or 7 or 8

10. exp Mental disease/
11. exp Mental Health/
12. Mental patient/
13. (mental* or psych*) adj2 (problem* or difficult* or disorder* or ill* or health*)
14. Schiz* or psychosis or psychotic or bi?polar or depress* or dysthimi* or mania or manic or neuros?s or psychoneuros?s or obsessive or compulsive or ocd or anxiety or ptsd or post?traumatic or panic or phobia
15. (delusional or paranoi* or mood or affective or neurotic or stress or reactive or combat or somatoform or somati?ation or phobic or adjustment or dissociat*) adj2 disorder*
16. Personality adj2 disorder*
17. eat* adj2 disorder*
18. Bulimi* or anorexi*
19. Bing* adj eat*
20. Compulsive adj (eat* or vomit* or purg*)
21. 10 or 11 or 12 or 13 or 14 or 15 or 16 or 17 or 18 or 19 or 20

22. 5 and 9 and 23

MATERNITY + INFANT CARE

1. Migration/ or asylum seekers/ or refugees/
2. refugee* or immigrant* or migrant* or immigration or migration or foreign* or new?comer*
3. asylum adj2 seek*
4. 1 or 2 or 3

5. Pregnancy/ or postnatal/ or birth/ or perinatal care/
6. pregnan* or post?partum or post?natal or puerperal or ante?natal or pre?natal or ante?partum or peri?natal or birth* or trimester*
7. 5 or 6

8. Mental disorders/ or mental health/
9. (mental* or psych*) adj2 (problem* or difficult* or disorder* or ill* or health*)
10. Schiz* or psychosis or psychotic or bi?polar or depress* or dysthimi* or mania or manic or neuros?s or psychoneuros?s or obsessive or compulsive or ocd or anxiety or ptsd or post?traumatic or panic or phobia
11. (delusional or paranoi* or mood or affective or neurotic or stress or reactive or combat or somatoform or somati?ation or phobic or adjustment or dissociat*) adj2 disorder*
12. Personality adj2 disorder*
13. eat* adj2 disorder*
14. Bulimi* or anorexi*
15. Bing* adj eat*
16. Compulsive adj (eat* or vomit* or purg*)
17. 8 or 9 or 10 or 11 or 12 or 13 or 14 or 15 or 16

18. 4 and 7 and 19

CINAHL

1. exp Emigration & Immigration/
2. exp Immigrants
3. exp Refugees
4. refugee* or immigrant* or migrant*or immigration or migration or foreign* or new?comer*
5. asylum n2 seek*
6. 1 or 2 or 3 or 4 or 5

7. exp Pregnancy/
8. exp perinatal care/ or exp postnatal period/ or exp pregnancy trimesters/ or exp pregnancy, multiple/
9. pregnan* or post?partum or post?natal or puerperal or ante?natal or pre?natal or ante?partum or peri?natal or birth* or trimester*
10. 7 or 8 or 9

11. exp Mental Disorders/
12. exp Mental Health/
13. Psychiatric patients/
14. (mental* or psych*) n2 (problem* or difficult* or disorder* or ill* or health*)
15. Schiz* or psychosis or psychotic or bi?polar or depress* or dysthimi* or mania or manic or neuros?s or psychoneuros?s or obsessive or compulsive or ocd or anxiety or ptsd or post?traumatic or panic or phobia
16. (delusional or paranoi* or mood or affective or neurotic or stress or reactive or combat or somatoform or somati?ation or phobic or adjustment or dissociat*) n2 disorder*
17. Personality n2 disorder*
18. eat* n2 disorder*
19. Bulimi* or anorexi*
20. Bing* n eat*
21. Compulsive n (eat* or vomit* or purg*)
22. 11 or 12 or 13 or 14 or 15 or 16 or 17 or 18 or 19 or 20 or 21

23. 5 and 9 and 23

COCHRANE

1. refugee* or immigrant* or migrant* or immigration or migration or foreign* or asylum
2. pregnan* or postpartum or postnatal or puerperal or antenatal or prenatal or antepartum or perinatal or birth or trimester
3. schiz* or psychosis or psychotic or bipolar or depress* or dysthimi* or mania or manic or neruosis or obsessive or compulsive or ocd or anxiety or ptsd or posttraumatic or post-traumatic or panic of phobia or bulimia or anorexia
4. #1 and #2 and #3
