# Supplementary material for: Prevalence and risk of mental disorders in the perinatal period among migrant women: a systematic review and meta-analysis
Source: Arch Womens Ment Health. 2017 Apr 8;20(3):449–62. doi: 10.1007/s00737-017-0723-z (PMC5423996; doi:10.1007/s00737-017-0723-z)
Supplement: Supplementary file 2 — (PDF 422 kb) [file 737_2017_723_MOESM2_ESM.pdf]

Table of studies

Name and year	Country	Study design and sample size	Mental disorder(s) and measurement(s)	Timepoint(s)	Inclusion and exclusion criteria, and other relevant information about sample	Prevalence and/or odds	Risk factors identified for migrant women and other relevant findings	Risk of selection bias	Risk of measurement bias
Abott 2006	New Zealand	Cross-sectional survey<br><br>1363	Depression<br>EPDS, >12	Postpartum<br>6 weeks	*Inclusion:* Children born at Middlemore Hospital, where the child had at least one parent who identified as being of Pacific Island ethnicity and was also a New Zealand permanent resident.	0.97 (0.72, 1.32)<br><br>16%	Whether born in New Zealand or not was not identified as a risk factor	M	M
Balbierz 2015	United States	RCT	Depression<br>EPDS, >9	Posptartum<br>3 month data used	*Inclusion:* All women 18 years or older, English or Spanish speaking, delivered infants with birthweights >=2,500g and had 5-min Apgar scores >6, and had a working telephone.<br><br>Majority of women were black or Latino, low-income sample.	1.13 (0.69, 1.85)<br><br>9%	Found significant differences in proportions of women scoring above cut-off on EPDS by race.	M	M
Balestrieri 2012	Italy	Cross-sectional survey<br><br>1608	Depression<br>EPDS, >12	Pregnancy<br>12-15 weeks	*Inclusion:* Women attending first ultrasound examination who were able to fill out EPDS and form in Italian<br><br>*Exclusion:* No Italian language, women with a cognitive deficit that hindered the reading and comprehension of questionnaires were excluded	0.82 (0.50, 1.35)<br><br>13%	Women born in other countries showed higher mean EPDS scores than Italian women	M	M
Ballantyne 2013	Canada	Cross-sectional survey<br><br>291	Depression<br>CES-D, >15	Postpartum<br>Week before discharge from NICU	*Inclusion:* All mothers who assumed primary responsibility for their infant and whose infant required neonatal intensive care unit (NICU) admission.<br><br>*Exclusion:* Non-English speaking, too ill, denied custody of infant	1.63 (1.01, 2.64)<br><br>59%	Immigrant mothers reported more depressive symptoms, poorer family functioning, less social support, and less mainstream acculturation. In final regression model immigrant status did not contribute to depressive symptoms.	M	M
Bandyopadhyay 2010	Australia	Cohort<br><br>10440	Depression<br>EPDS, >12	Postpartum<br>6 months	Only included migrants from non-English-speaking countries<br><br>"As the findings for immigrant women from English-speaking countries proved very similar to those of Australian-born women, their experiences are not reported in this paper"	1.38 (1.13, 1.69)<br><br>20%	Immigrant mothers less proficient in English had raised odds<br>of scoring as probably depressed on the EPDS, compared with Australian-born women (28.8% vs 15%, OR 2.40, 95% CI 1.9- 3.0). No significant difference was observed between immigrant women proficient in English and Australian- born women (15.9% vs 15%, OR 1.07, 95% CI 0.7-1.5). The proportions of women scoring as depressed in	M	M

							the sample<br>varied by maternal country of birth: Malaysia (40%), India (39%), Italy (31%), Vietnam (24%), China (23%) and the Philippines (19%), followed by women born in Greece (16%), Sri Lanka (15%), Germany (7%) and the Netherlands (6%).		
Bjerke 2008	Norway	Cohort<br><br>197	Depression<br>EPDS, >9	Postpartum<br>6 to 12 weeks	*Inclusion:* Pakistan women living in Oslo or one of the suburbs	8%	Higher score on life events, history of prior depression, being single and an age over 30 years were risk factors for depression.	H	M
Chen 2012	Taiwan	Cross-sectional survey<br><br>190	Depression<br>EPDS, >9	Postpartum<br>Up to 1 year	*Inclusion:* Immigrant mothers from China and Vietnam to Taiwan who gave birth and registered at the Health Centers of Beitou and Chilin districts within 1 year of delivery	42%<br><br>Chinese: 30%<br><br>Vietnamese: 57%	“Doing-the-month” practices was negatively associated with PPD symptoms. Immigrant mothers from Vietnam had higher odds of PPD than those from China.<br><br>Women in Taiwan adhere to traditional “doing the month” practices. Women frm China have similar postpartum practices, whereas women from Vietnam have somewhat differed posptartum cultural practices.	H	M
Chen 2013	Taiwan	Cohort<br><br>203	Depression<br>EPDS, >9	Postpartum<br>1 and 6 months	*Inclusion:* Immigrant women married to Taiwanese men from China or Vietnam who just gave birth to a child and lived in the five districs of Taipei City. Registered at the five centres and >18 years old	24% at 1 month<br><br>12% at 6 months	Decrease in prevalence of depression from 1 – 6 months was significant. Mean social support scores did not differ between the two timepoints.<br><br>Negative and significant relationship between social support and postpartum depression among immigrant women.<br><br>Immigrant women who had greater social support and fewer depression symptoms at 1 month also had better social attitudes.	H	M
Chien 2012	Taiwan	Cross-sectional survey<br><br>190 Migrant, 190 non-migrant	Depression<br>EPDS, >12	Postpartum<br>Up to 1 year	*Inclusion:* Married, immigrant women from China or Vietnam who had given birth to healthy newborns during the past 2 years and were willing to participate in the study<br>Native women who had given birth to healthy newborns in past 2 years, recruited to match the frequency distribution with respect to months after delivery among immigrant sample.<br>For this study only women in first year postpartum included	5.2 (2.6, 10.4)<br><br>24%	Domestic decision-making power and social support were significant and independent protective factors against postpartum depression symptoms among immigrant and native women in Taiwan, after adjustment for background variables. Immigrant mothers had a higher rate of postpartum depression symptoms and lower social support than native mothers. Perceived family income sufficiency was also associated with depression symptoms.	H	M
Choi 2012	South Korea	Cross-sectional survey<br><br>141 analysed	Depression<br>EPDS, >12	Postpartum<br>Up to 3 months	*Inclusion:* Korean mothers and married immigrant women from Vietnam who live in medium-sized areas of South Korea, and who gave birth less than 3 months prior to the time of data collection<br><br>*Exclusion:* History of depression, prenatal or post-partum complications	2.42 (1.11, 5.27)<br><br>35%	Immigrant Vietnamese mothers had a significantly higher rate of PPD than native Korean mothers.	H	M

Connelly 2013	United States	RCT (data from screening phase)<br><br>1868	Depression EPDS, >9	Anytime throughout the perinatal period at routinely scheduled visits, including the 6-week postpartum visit (combined pregnancy and postpartum)	*Inclusion:* Women were eligible if they were pregnant, English or Spanish speaking, and reachable by phone.<br><br>*Exclusion:* Being a surrogate mother or having a cognitive impairment precluding ability to give informed consent and respond to psychosocial questionnaires. Minors who were receiving reproductive health services were considered to be emancipated and thus able to provide legal consent to participate.<br><br>82% of women were Latina, and 55% were born outside the US. Mainly low-income Mexican women.	0.77 (0.61, 0.96)<br><br>18%	The mothers who screened positive for depressive symptoms reported significantly more IPV and substance use problems than those screening negative.	L	M
Davila 2009	United States	Cross-sectional survey<br><br>455	Depression CES-D, >20	Anytime in pregnancy and up to one year postpartum	*Inclusion:* Pregnant and postpartum women (up to one year postpartum), that attended any one of the SAMHD's family planning or prenatal care clinics and self-identified as Latina.<br><br>*Exclusion:* women born in Central and South American countries so as to compare Latinas born in the U.S. and Mexico	0.56 (0.34, 0.91)<br><br>18%	Birth country, marital status and pregnancy status were significant predictors. Single women were three times more likely than married women and pregnant women were 2.5 times more likely to have a CES- D score of 21 or greater. Mexican-born women were significantly less likely to express moderate to high levels of depressive symptoms compared to U.S.-born women.<br><br>When higher cut-off of 24 or more on CES-D was used, country of birth was no longer a significant predictor of depressive symptoms.	H	M
Dennis 2004	Canada	Cross-sectional survey<br><br>594	Depression EPDS, >9	POST 1W	*Inclusion:* Over 18, able to understand English	2.7 (1.45, 4.85)	In the multivariate model, only immigration within the last 5 years remained in the final model among sociodemographic variables.<br><br>Immigration within last 5 years unadjusted OR: 8.46 (2.29, 31.19)<br><br>Adjusted: 4.94 (0.99, 24.8)	M	M
Diaz 2007	United States	Cohort<br><br>69	Depression CES-D, >15	Data given for pregnancy and 6 month postpartum time-points	*Inclusion:* Low-income, pregnant Latinas receiving prenatal care in a hospital in Northern California. Between 15-30, English or Spanish speaking and in second trimester of pregnancy, identifying as Latina and being in an intimate relationship<br><br>*Exclusion:* Current diagnosis of major depressive disorder, substance use disorder, experiencing a psychotic episode, a serious medical condition, and/or other psychosocial problems, such as	Pregnan cy: 0.26 (0.04, 1.79)<br><br>28%<br><br>Postpart um: 0.29		H	M

					homelessness	(0.04, 2.36)<br><br>23%			
Eastwood 2011	Australia	Cross-sectional survey<br><br>25455	Depression EPDS, >12	First child and family nurse visit (mean 2.16 weeks), up to 3 months postpartum	*Inclusion:* Mothers of infants born in South West Sydney who received a first visit from a child and family nurse within 3 months of birth<br><br>*Excluded:* Not completed EPDS	Unadjusted: 1.9 (1.7, 2.1)<br><br>Adjusted: 1.6 (1.4, 1.8)	Adjusted for: Accomodation, financial situation, mother's highest education, marital status, car access, suburb duration, sorry to leave suburb, current breast feeding, health rating of self, planned pregnancy, health rating of child	M	M
Elo 2010	United States	Baseline survey of a prospective cohort study<br><br>3101	Depression CES-D, >22	Pregnancy up to 21 weeks	*Inclusion:* Spoke English or Spanish, lived within City of Philadelphia and were less than 21 weeks gestation at first prenatal care visit<br>Limited the sample to women who self-identified as Black or African American and who were born in the United States, the Caribbean, or Africa.	1.24 (0.94, 1.65)<br><br>26%	Foreign-born Black women were significantly less likely than native-born Black women to engage in negative health behaviours and more likely than were native-born Black women to report good physical and mental health.	M	M
Escribà-Agüir 2013	Spain	Cohort<br><br>1309	Depression EPDS, >10	Pregnancy (1st trimester), postpartum 5 months and 12 months. Raw data only available for 1st trimester.	*Inclusion:* Women attending one of nine primary care centres of the Valencia Region (East Spain) during their first trimester of pregnancy<br><br>*Exclusion:* Unable to complete the self-administered questionnaire, either because they were illiterate or did not understand Spanish. Females who attended the practice with a male partner were also considered ineligible (as the study was looking at IPV).	3.42 (2.49, 4.70)<br><br>29%		M	M
Fisch 1997	Israel	Cross-sectional survey<br><br>327	Depression EPDS, >8	Postpartum 6-12 weeks (also 2-3 days post-delivery)	*Inclusion:* Women registering in the maternity department<br><br>*Exclusion:* Women who were unwilling to participate in the study, those who spoke insufficient Hebrew to fill in the questionnaire, those who did not intend to return for the routine postpartum gynaecological check-up in our hospital	2.51 (1.23, 5.13)<br><br>18%		H	M
Fleuriet 2015	United States	Cross-sectional survey<br><br>631	Depression PHQ-9, >9	Pregnancy any time	Low income pregnant women aged 18 or over whose clinicians had determined they had low medical risk for having low birth weight infants. Participants were born in Mexico or self-identified as Mexican American, born in the US.	0.7 (0.43, 1.12)<br><br>80%	Mexican immigrant women had statistically significant lower scores in perceived social stress and depression, lower scores in subjective social status and self-esteem, and higher scores in pregnancy-related anxiety than Mexican American	H	M

							women. There were not significant differences in marital status, medical problems with pregnancies, gestational age at the time of the survey and gestational age when prenatal care.<br><br>Scores of psychosocial measures such as depression, pregnancy related anxiety and perceived stress significantly correlated with years lived in the U.S. Depression level increased, pregnancy related anxiety decreased and perceived stress increased as the number of years lived in the U.S increased		
Fortner 2011	United States	Cohort<br><br>921	Depression EPDS, >12	Pregnancy Up to 20 weeks gestation	*Inclusion:* Women of Puerto Rican or Dominican Republic heritage<br><br>*Exclusion:* Current medications which adversely influence glucose tolerance, multiple gestation, history diabetes, hypertension, heart disease or chronic renal disease, and <16 years >40 years of age.	0.84 (0.63, 1.12)<br><br>29%	Looked at "generation in the continental US, e.g. first, second and third generation".<br><br>Women who preferred English or were second generation in the US were more likely to report depressive symptoms compared to less acculturated women.	H	M
Gagnon 2013	Canada	Cohort<br><br>1184	Depression EPDS > 9<br><br>PTSD Reported "a lot" or "extremely" to one or more trauma symptom(s) in the last week on the Harvard Trauma Questionnaire	Postpartum 4 months (and 1 week)	*Inclusion:* (1) planned to remain in the study city to 16 weeks post-partum; (2) were able to speak any of the study languages [Arabic, Dari/ Persian, English, French, Mandarin/Cantonese (oral; 'simplified' and 'complex' Chinese written), Punjabi, Russian, Serbo-Croatian, Somali, Spanish, Tamil and Urdu]; (3) lived within a 45-min drive of the hospital; (4) were discharged from hospital by post-partum day 4 for vaginal or day 7 for caesarean births; and (5) met the criteria for one of the following study subgroup samples (refugees, asylum-seekers, immigrants, and Canadian-born based on their status in Canada).<br><br>All migrant groups were restricted to those living in Canada <5 years.<br><br>*Exclusion:* Women were excluded if they (1) had a major mental illness (schizophrenia, other psychoses or profound previously existing depression) or cognitive impairment that precluded informed consent; (2) were temporary residents (e.g. visitors, students); (3) had given the infant up for adoption; or (4) had a stillbirth delivery or an infant death.	Depression: 2.87 (1.81, 4.54)<br><br>23%<br><br>PTSD: 17%	By migrations status:<br><br>EPDS>9<br>Refugees: 17%<br>Asylum-seekers: 23%<br>Immigrants: 14%<br>Canadian born: 7%<br><br>PTSD:<br>Refugees: 34%<br>Asylum-seekers: 48%<br>Immigrants: 15%<br>Canadian born: not reported<br><br>International migrants from a range of countries experienced a greater number of professionally-identified concerns post-birth and were less likely to have those concerns addressed by the health care system than Canadian-born women.	M	M
Ganann	Canada	Cross-	Depression	Postpartum	*Inclusion:* woman of ≥16 years of age; delivery of a	1.77	Immigrant women perceived health services less	L	M

2012		sectional survey<br><br>1047	EPDS, >11	6 weeks	live singleton infant; gestational age ≥37 weeks; mother assuming care of infant when discharged; mother competent to give consent; and mother can be contacted by telephone.<br><br>Women were ineligible to participate if their infant required admission to a neonatal intensive care or special care nursery for more than 24 hours or were unable to communicate in one of the four study languages (English, French, Chinese, Spanish).	(1.07, 2.92)<br><br>11%	favorably than their Canadian-born counterparts and were more likely to have unmet service needs for both physical and emotional health problems.		
Glasser 2000	Israel	Cohort<br><br>288	Depression EPDS, >9	Pregnancy 26 weeks, and postpartum 6 weeks<br><br>(EPDS only postpartum)	*Inclusion:* Random sample of every second woman consecutively registering at the major prenatal care clinic in a low socioeconomic level community in central Israel.<br><br>*Exclusion:* Those who aborted, miscarried, or moved out of the community before the 26th week of pregnancy were excluded from the study.	2.35 (1.34, 4.12)<br><br>32%	Russian new immigrant status was significantly related to PPD.<br><br>For new Russian immigrants, marital disharmony, history of emotional problems and maternal age <19 were risk factors for PPD, however poor social support, pregnancy depression and ill baby which were risk factors for Israeli-born women were not risk factors for new Russian immigrants.	M	M
Goyal 2006	United States	Cross-sectional survey<br><br>58	Depression PDSS (Postpartum Depression Screening Scale), >80	Postpartum between 2 weeks and 12 months	*Inclusion:* Immigrant Asian Indian women, at least 18 years old, able to read and write in English + between 2 weeks and 12 months postpartum, and had given birth to a live healthy infant	24% (>80), 28% minor postpartum depressive symptomatology (60-79)	There were no statistically significant differences in the level of postpartum depressive symptomatology between women in arranged and non-arranged marriages.	H	M
Harrison 2009	United States	Cross-sectional survey<br><br>1492	Depression PHQ-9, >9	Pregnancy At prenatal intake appointment	*Inclusion:* All women attending the clinics<br><br>*Exclusion:* Women with incomplete data on substance misuse variables were excluded (24 women)<br><br>The study sample included predominantly women of colour.	0.55 (0.40, 0.74)<br><br>13%	Much lower rates of pre-pregnancy substance use among immigrants compared with their U.S.-born racial or ethnic counterparts.	L	M
Heilemann 2004	United States	Cross-sectional survey<br><br>129	Depression CES-D, >15	Mixed sample of pregnant and postpartum women<br>59 pregnant	*Inclusion:* Women of Mexican descent, between 21-40, who could read and write in Spanish or English were eligible for larger study - those who were pregnant or 6 months postpartum were included in this analysis<br><br>*Exclusion:* Not perinatal (pregnancy and 6m post)	Pregnancy: 0.34 (0.10, 1.15), 45%	Language and birthplace were not useful indicators of intragroup differences for this sample, exposure to the US in childhood was. Early exposure to the US was associated with deleterious effects on women's health and well-being related to depressive symptoms.	H	M

				70 <6 months postpartum		Postpartum: 0.44 (0.17, 1.17), 50%			
Huang 2007	United States	Cohort<br><br>7676	Depression CES-D, >14 (only reporting severely depressed)	Postpartum 6 months up to one year.	The target population of the ECLS-B consists of all children born in the U.S. in the year 2001 except the follow- ing: (1) children born to mothers less than 15 years of age; (2) children who died before the 9-month assessment; and (3) children who were adopted prior to the 9-month assessment.	0.75 (0.59, 0.94)<br><br>5%	For non-Hispanic whites, no significant difference was found in the prevalence of depressive symptoms by nativity. Among non-Hispanic Blacks, com- pared to their US-born counterparts, foreign-born mothers had lower prevalence of depressive symptoms at mild (20.7% vs. 30.6%) and severe categories (4.9% vs. 10.7%). Among Hispanics, foreign-born Hispanic mothers had a lower prevalence in every category of depressive symptoms. The overall pattern in Asian mothers was the opposite to that of Hispanic mothers. For most foreign-born Asian mothers (Asian Indian, Chinese, Filipina, Japanese, and Other), their prevalence of any depressive symptom was higher than US-born Asians. Specifically, foreign-born Japanese mothers had significantly higher rates of having some depressive symptoms than those who were US-born	L	M
Huang 2008	Taiwan	Cross-sectional survey<br><br>106	Depression EPDS, >12	Postpartum 6 months	*Inclusion:* Vietnamese and Indonesian women living in Taiwan for more than 1 year, 6 months post-uncomplicated delivery, conversational ability in Taiwanese and/or Mandarin Chinese	25%	No associations were found between the sociodemographics of the women and scores on the EPDS. However, a statistically significant association was found between a score of 13 or more and self-reported “emotional distress” following birth.	H	M
Hung 2012	Taiwan	Cross-sectional survey<br><br>340	Depression BDI-II, >19 (moderate-severe depression cut-off)	POST	*Inclusion:* Postpartum immigrant women who were married and could communicate in Mandarin Chinese	0.6%		H	M
Jayaweera 2010	UK	Cohort<br><br>17258	Depression Modified malaise inventory score, >3 (classified as depressed/a	Postparum Around 9 months	*Inclusion:* Infants selected from child benefit records<br><br>*Exclusion:* Families not eligible for universal benefits such as those with legal statuses that preclude them from access to public funds and services were not eligible for the MCS. Among the excluded are those most likely to face barriers in	Unadjusted: 1.14 (0.99, 1.31)<br><br>15%	Adjusted for ethnic group/country of birth, parity, age, education, occupational class, ward type, lone parent.<br><br>In the adjusted analysis, mothers born abroad were less likely to be depressed than UK born mothers, whereas mothers in Other White, Indian and Pakistani ethnic groups were significantly	L	M

			nxious)		accessing free healthcare, such as undocumented migrants and failed asylum seekers.	Adjusted OR: 0.79 (0.62-1.00)	more likely to be depressed than White British/Irish mothers irrespective of country of birth and demographic and socio-economic differences (Table 2). The adjusted analysis of migrant mothers showed a significant linear trend of increasing depression with length of residence in the and more depression among mothers in Other White, Indian, Pakistani and Black Caribbean ethnic groups compared with White British/Irish migrant mothers.		
Lanes 2011	Canada	Cross-sectional survey<br><br>6421 (but weighted to represent 76509 Canadian women)	Depression EPDS, >12	Postpartum Up to 1 year	*Inclusion:* Aged 15 years of age or over, had delivered a live singleton infant, and were living with their infant at the time of the interview<br><br>*Exclusion:* Women living on First Nations reserves or in institutions at the time of the survey were excluded	Unadjusted: 2.71 (2.20, 3.32)<br><br>Adjusted: 2.35 (1.77-3.13)	Adjusted for socioeconomic factors, demographic factors, maternal characteristics including previous diagnosis of depression, stress levels during pregnancy and other variables including availability of support after pregnancy and planned pregnancy.	L	M
Luecken 2013	United States	Cohort<br><br>320	Depression EPDS, >12	Pregnancy and six weeks postpartum (raw data only available for pregnancy timepoint)	*Inclusion:* Self-identification as Mexican or Mexican-American, fluency in English or Spanish, age 18 or older, low-income status (family income below $25000 or eligibility for Medicaid or Federal Emergency Services) and no prenatal evidence of a serious infant health or developmental problem<br><br>*Exclusion:* Reported not being married or in romantic relationship at prenatal visit<br><br>89% of the sample was foreign born.	0.54 (0.24, 1.23)<br><br>12%		H	M
McDonald 2013	Canada	Cohort<br><br>3388	Depression EPDS, >12	Pregnancy Less than 25 weeks gestation and 4 months postpartum	*Inclusion:* Less than 24+6 gestation at time of recruitment, 18+, receiving prenatal care in Calgary, able to complete questionnaires in English	Pregnancy: 2.26 (1.74, 2.94) 14%<br><br>Postpartum: 1.19 (0.81, 1.75) 6%		M	M

Mechakra-Tahiri 2007	Canada	Cross-sectional survey<br><br>1875	Depression CES-D, >12	Postpartum Around 5 months	*Inclusion:* All 1998 singleton live births were considered for eligibility<br><br>*Exclusion:* Babies born before 24 or after 42 gestational weeks, those with unknown gestational age, those born in Cri and Inuit territories, on Indian reservations or in the Northern region of Quebec.	2.05 (1.45, 2.90)<br><br>21%	Depressive symptoms among immigrant women belonging to minority groups (24.7%) were higher than among women born in the host country (11.2%) and among immigrant women belonging to majority ethnocultural groups (8.3%).	M	M
Milgrom 2008	Australia	Cohort<br><br>12361 (10907 with country of birth)	Depression EPDS, >12	Pregnancy and 6 weeks postpartum (raw data available for antenatal only)	*Inclusion:* Basic ability to read and understand written English	1.02 (0.85, 1.22)<br><br>7%		M	M
Minde 2001	Canada	Cross-sectional survey<br><br>42	Depression EPDS, >12	POST (around)	*Inclusion:* Full-term infants, Apgar score at least 6 and 8 at 1 and 5 mins, birthweight>2500g, and no congenital abnormalities. Parents had to speak either English or French and had no prior contact with the physician they had chosen to care for their infant	2 (0.39, 10.34)<br><br>22%		H	M
Miszkurka 2010	Canada	Cross-sectional survey<br><br>5162	Depression CES-D, >15	Pregnancy 24-26 weeks	*Inclusion*: Aged 18 or over at expected delivery date, singleton gestation and fluency in English or French.<br><br>*Exclusion*: Women presenting severe chronic illness, placenta previa, cervical incompetence diagnosed in previous pregnancy, impending delivery, or a fetus affected by a major anomaly	1.59 (1.39, 1.82)<br><br>32%	For all women: marital strain, lack of social support, lack of money for two or more basic needs, and life event distress were all strongly and independently associated with AD. A greater likelihood of presenting AD while lacking money for basic needs was observed among newly arrived women. The likelihood of presenting AD while lacking social support was highest among Canadian-born women. High marital strain was associated with AD in Canadian born and intermediate-term immigrant women. Adverse life event distress was more strongly associated with AD in newly arrived immigrant women than in any other group. At the same level of exposure to these contextual risk factors, Canadian-born women presented higher vulnerability to AD when lacking social support while immigrant women presented higher vulnerability to AD when lacking money for basic needs.<br>Age-adjusted OR in relation to region of origin showed a twofold likelihood of DS in women from the Caribbean, Maghreb and Sub- Saharan Africa, when compared to Canadian women.	M	M
Peer 2013	Canada	Cross-sectional	Depression EPDS, >11	Pregnancy 19 weeks	*Inclusion:* Women who self-identified as being born outside of Canada, with a singleton	17%	Depressed women reported more perceived stress, more somatic symptoms, lower social	H	M

		survey<br><br>78			pregnancy of less than 27 weeks gestation, were recruited from the community<br><br>*Exclusion:* Current PTSD		support, and were less often working or attending school during pregnancy.		
Ratcliff 2015	Switzerland	Prospective cross-sectional<br><br>228 (93 w EPDS)	Depression EPDS, >11	Pregnancy 3rd trimester	*Inclusion:* All immigrant women attending a programme of antenatal classes for immigrant women with low French proficiency. Translations of EPDS and interpreters used if needed.	37%	Psychosocial risk factors: women facing several disadvantages simultaneously (i.e. precarious legal status, lack of marital support and housing difficulties) scored higher on EPDS. Lack of marital support, and short duration of residence in the host country, increased the risk of depressive symptomatology.	H	M
Rudman 2008	Sweden	Longitudinal survey<br><br>2338	Depression EPDS, >12	Pregnancy (first booking visit) and postpartum (2 months)	*Exclusion:* Those who had a miscarriage, attended non-participating clinics, did not speak Swedish	Pregnancy: 2.36 (1.72, 3.24), 20%<br><br>Postpartum: 2.45 (1.64, 3.64), 14%		M	M
Shafiei 2015	Australia	Cross-sectional survey<br><br>39	Depression EPDS, >12	Postpartum 4 months	*Inclusion:* Immigrant women who were born in Afghanistan, spoke Hari/Persian, and had given birth to a live and healthy baby, and if their own health did not warrant concern	31%		H	M
Small 2003	Australia	Cross-sectional survey<br><br>318	Depression EPDS, >12	Postpartum 6-9 months	*Inclusion:* All women born in Vietnam, Turkey and the Philippines who had given birth to a live baby over 1500g, and whose own health and that of the baby didn't warrant concern	15%	Social support, physical health, facility with English, length of residence, reason for migration were associated with depression.	M	M
Stewart 2008	Canada	Cross-sectional survey<br><br>341	Depression EPDS, >9	Postpartum 7-10days	*Inclusion:* Women giving birth in included hospitals who spoke any of the study languages. People in migrant group were newcomers (<5 years in Canada).	5.1 (2.13, 12.22)<br><br>31%	EPDS>9 was predicted by immigrant, asylum seeker, refugee, and visible minority group but not related to number of years living in Canada, living alone, living with the infants father, education, number of living children, specialist obstetrical care, labour support by a doula, attendance at antenatal clinic, low income, normal examination of the infant, male infant, physical abuse, or forced sex.<br><br>All groups of newcomers with elevated EPDS scores had lower social support scores than Canadian-born women.	M	M

Stewart 2012	Canada	Cohort<br><br>774	Depression EPDS, >9	Posptartum 4 months	*Inclusion:* Pregnant migrant women, in Canada <5 years, who were refugees, asylum seekers or nonrefugee immigrants. Women had to be discharged by day 4 and day 7 postbirth for vaginal and cesarean deliveries, respectively.	19%	Abused migrant women were more likely to report a score above the cut-off on the EPDS than nonabused women	M	M
Sword 2006	Canada	Cross-sectional survey<br><br>1250	Depression EPDS, >11	Postpartum 4 weeks	*Inclusion:* Vaginal birth to single live infant, were being discharged from hospital at the same time as their infant, were assuming care of their infant at the time of discharge, and were competent to give consent.<br><br>*Exclusion:* Infant who required admission to a neonatal intensive care or special care nursery for more than 24 hours or were unable to communicate in one of the four study languages (English, French, Spanish & Chinese)	2.27 (1.44, 3.58)<br><br>15%		M	M
Tran 2002	Australia	Cohort<br><br>829	Depression EPDS, >12	Pregnancy and postpartum combined (perinatal)	*Inclusion:* Ethnic women (Arabic, Cambodian, Chinese, Laotian and Vietnamese) accessing the EOLO (Ethnic Obstetric Liaison Services)	7%		M	M
Tsao 2015	Taiwan	Cohort<br><br>44	Depression EPDS, >12	Pregnancy and postpartum (6 weeks)	*Inclusion:* Of Vietnamese descent and recently immigrated to Taiwan (less than three years ago), at least 18 years of age, expected to give birth to a live, singleton baby, and a legal resident<br><br>*Exclusion:* Women with current or past mental health problems, or who subsequently had a stillbirth, neonatal death, and live births with a congenital anomaly were excluded	Pregnancy: 32%<br><br>Postpartum: 21%	Maternal antenatal depression (EPDS) was significant and positively related to psychological distress scores on the GHQ-12, and DASS subscales.<br><br>Mothers with a low birth weight infant were more likely to report higher levels of depression. Women reporting higher levels of difficult life circumstances, less social support (SSA), as well as more child-care stress (CCSC) and more psychological distress (GHQ-12, DASS-A,DASS-S), were more likely to suffer from postnatal depression.	H	M
Valentine 2011	United States	Cohort<br><br>210	Depression BDI-FS, >3<br><br>Beck's Depression Inventory – Fast Screen	Postpartum Up to 1 year	Inclusion: 18+, self-identified as Latina, over 12 weeks pregnant, not planning to move in next 12 months, planning to raise child themselves.	1.49 (0.77, 2.88)<br><br>51%	In whole sample (majority migrant) prenatal predictors of PPD included depression, recent IPV exposure, remote IPV exposure, non-IPV trauma history, poverty, low social support, acculturation, high parity, and low education.<br><br>Recent IPV, prenatal depression, non-IPV trauma, and low social support were associated with greater likelihood of PPD in bivariate analyses. Recent IPV and prenatal depression continued to show significant association with PPD in	H	M

							multivariate analyses, with greater odds of PPD associated with recent IPV than with prenatal depression		
van Lieshout 2011	Canada	Cohort<br><br>656	Depression 15-item version CES-D that emerged from study measurement invariance analyses, >13	Postpartum 2 months	*Inclusion:* New mothers giving birth to infants in the cities of Toronto and Hamilton between April 2006 and September 2001. Eligible participants also had to have at least one other child under 16 years of age in addition to their newborn.<br><br>*Exclusion:* Unable to speak English<br><br>Individuals born in a country other than Canada who had immigrated within the last 10 years or who identified themselves as a refugee. Individuals who were born in Canada, the US the UK or Australia or in another country but who had lived in Canada for more than 10 years comprised the non-immigrant group/	Unadjusted: 1.65 (1.04, 2.61),20 %<br><br>Adjusted: 2.16 (1.10-4.19)	Immigrant status, younger maternal age, being a single mother, having a household income below the LICO, fewer years of education, increased conflict with one's partner, difficult pregnancy, past history of abuse or sexual abuse all predicted higher levels of depressive symptoms as well as an increased risk of being a potential case of depression. Maternal smoking and English not being the primary language spoken in the home predicted higher levels of depressive symptoms, but not depression case status.	M	M
Yelland 2010	Australia	Cross-sectional survey<br><br>4366	Depression and Anxiety DASS (The Depression Anxiety Stress Scales), >9 (depression) , >7 Anxiety	Postpartum 6 months	*Inclusion:* All women who gave birth in a four week period in Victoria and an eight week period in South Australia in September/October 2007<br><br>*Exclusion:* Those who had a stillbirth, or whose baby was known to have died.	Depression: 1.1 (0.89, 1.35) ,18%<br><br>Anxiety: 1.28 (1.01, 1.62), 15%	Only maternal country of birth remained a significant predictor of depression at the multivariate level.<br><br>Divided migrant group into English-speaking and non-English-speaking background. For migrant women with NESB significantly higher risk of depression and anxiety.<br><br>There was a high level of comorbidity – women experiencing anxiety and depression.	H	M
Yeung 1986	United States	Cross-sectional survey<br><br>124	DSM-III diagnosis (Adjustment disorder with depressed mood, Adjustment disorder with anxious mood, Dysthymic disorder, Psychological factors	Pregnancy	*Inclusion*: Chinese pregnant women, all planning on carrying pregnancy to term, all married and living with spouse<br><br>*Exclusion*: Alcohol or drug abuse	23%		H	L

			affecting physical condition, PTSD)<br>Psychiatric interview						
Yoshida 1997	UK	Cohort<br><br>98	Depression<br>Psychiatric interview by Japanese psychiatrist using translation of Schedule for Affective Disorders and Schizophrenia (SADS) and Research Diagnostic Criteria (RDC) diagnoses, NA	Postpartum<br>Within 3 months	*Inclusion:* Pregnant Japanese women living in London	12%	Depression was associated with having had a stressful life event or obstetric difficulty but without grandmothers' support – depressed and non-depressed women were equally likely to have had their mothers visit England to attend the delivery.<br><br>Sensitivity of EPDS was only 25%, even at lower cut-off suggested for Japanese women.	H	M
Zelkowitz 2008	Canada	Cohort<br><br>119	Depression<br>EPDS, >11	Pregnancy and postpartum	*Inclusion*: Born outside Canada or the US, age 18 or older, immigrated to Canada as an adult, or with a conjugal partner, able to respond to questions in English French or Spanish	Pregnancy: 42%<br>Postpartum: 38%	Prenatal depressive and somatic symptoms, as well as marital quality, were the best predictors of postpartum depressive symptomatology. Women with relatively few somatic complaints, low levels of perinatal stress, and satisfactory marital relations were less likely to exhibit mental health problems during pregnancy and postpartum. Women who were not depressed prenatally but reported postpartum depressive symptomatology exhibited several predisposing risk factors during pregnancy: many somatic complaints, high perinatal anxiety, and pre-migration stress. Women who were depressed during pregnancy but not postpartum reported improved physical function after childbirth.	M	M
